# Highly phosphorescent platinum(ii) emitters: photophysics, materials and biological applications†
†Electronic supplementary information (ESI) available. See DOI: 10.1039/c5sc03766b



**DOI:** 10.1039/c5sc03766b

**Published:** 2016-01-07

**Authors:** Kai Li, Glenna So Ming Tong, Qingyun Wan, Gang Cheng, Wai-Yip Tong, Wai-Hung Ang, Wai-Lun Kwong, Chi-Ming Che

**Affiliations:** a State Key Laboratory of Synthetic Chemistry , Institute of Molecular Functional Materials , HKU-CAS Joint Laboratory on New Materials and Department of Chemistry , The University of Hong Kong , Pokfulam Road , Hong Kong , China . Email: cmche@hku.hk; b HKU Shenzhen Institute of Research and Innovation , Shenzhen 518053 , China

## Abstract

In recent years a blossoming interest in the synthesis, photophysics and application of phosphorescent Pt(ii) complexes, particularly on their uses in bioimaging, photocatalysis and phosphorescent organic light-emitting diodes (OLEDs), has been witnessed. The superior performance of phosphorescent Pt(ii) complexes in these applications is linked to their diverse spectroscopic and photophysical properties, which can be systematically modulated by appropriate choices of auxiliary ligands. Meanwhile, an important criterion for the practical application of phosphorescent metal complexes is their stability which is crucial for biological utilization and industrial OLED applications. Taking both the luminescence properties and stability into consideration, chelating ligands having rigid scaffolds and with strong σ-donor atoms are advantageous for the construction of highly robust phosphorescent Pt(ii) complexes. The square-planar coordination geometry endows Pt(ii) complexes with the intriguing spectroscopic and photophysical properties associated with their intermolecular interactions in both the ground and excited states. In this article, we discuss the design and synthesis of phosphorescent Pt(ii) complexes with elaboration on the effects of ligands on the structure and luminescence properties. Based on their photophysical and emission properties, we intend to shed light on the great promise of highly robust phosphorescent Pt(ii) emitters in an array of applications from molecular materials to biosensors.

## Abstract 2

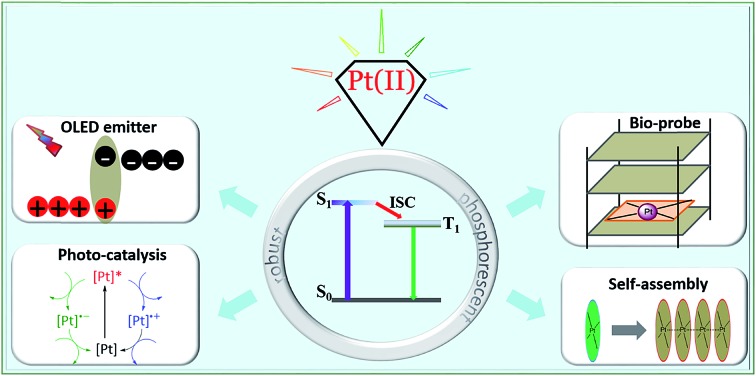
The structural effects of ligands on the emission properties of Pt(ii) complexes and promising applications of luminescent Pt(ii) complexes in various areas are discussed.

## 1 Introduction

**Fig. 1:**
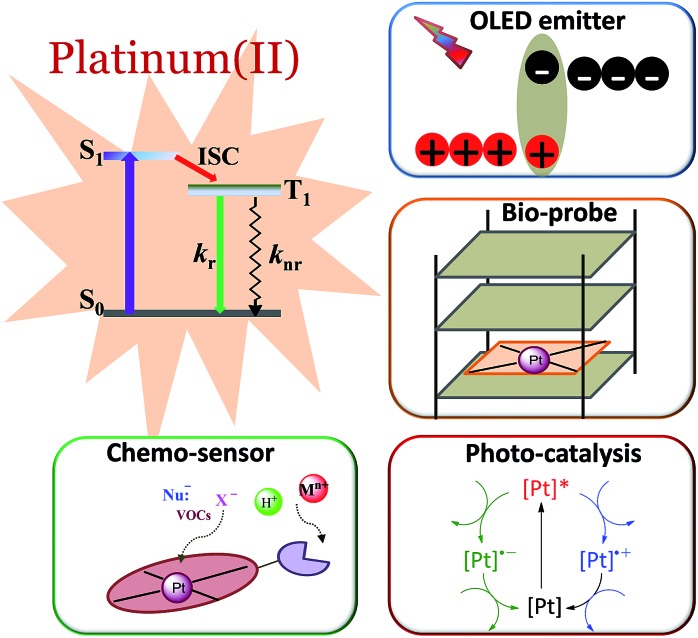
Various applications of phosphorescent Pt(ii) complexes.

Phosphorescent transition metal complexes are distinct from pure organic luminophores due to their characteristic long emission lifetime, large absorption–emission Stokes shift, and tuneable excited states. The efficient phosphorescence from transition metal complexes at room temperature is attributed to the heavy atom effect that induces strong spin–orbit coupling (SOC), facilitating both fast intersystem crossing (ISC) and the formally spin-forbidden triplet radiative decay.^1,2^ In the literature, there are numerous reports on the photophysical and photochemical properties of transition metal complexes, particularly those of Ru(ii), Ir(iii), and Pt(ii).^3–7^ Platinum, being a third-row transition element, has the second largest SOC constant. In contrast to d^6^ Ru(ii) and Ir(iii) complexes that have an octahedral coordination geometry, d^8^ Pt(ii) complexes usually adopt a square planar coordination geometry with open axial coordination sites allowing for structural distortion, inner sphere substrate binding, and intermolecular interactions, all of which can significantly alter the ground state and excited state properties.^8^ We and others have been working to develop new classes of phosphorescent Pt(ii) complexes and explore their applications in luminescent chemosensing,^9–12^ photocatalysis,^13–16^ optical limiting,^17^ bioimaging,^18–21^ and organic light-emitting diodes (OLEDs)^22^ (Fig. 1).

State-of-the-art phosphorescent OLEDs based on Ir(iii) and Pt(ii) emitters have exhibited high efficiencies with an external quantum efficiency (EQE) > 20%. However, one of the obstacles in the commercialization of phosphorescent OLEDs is the stability of the various materials used in the device fabrication. Hence, a high robustness of phosphorescent transition metal complexes is essential for enabling their practical application. In this regard, triplet emitters should be (1) tolerant to thermal deposition during device fabrication, (2) resistant to structural rearrangement as encountered in some Ir(iii)-OLED emitters during thermal treatment^23–25^ and (3) stable against chemical degradation that leads to device operational aging.^26^ As bioimaging agents, the phosphorescent metal complexes should preferably be kinetically inert against ligand-exchange reactions or biological reduction under physiological conditions.^18^ In photocatalysis, the metal complex acting as the photosensitizer would have to be stable against photobleaching and solvent-induced decomposition in order to afford a high product turnover.^27^

The robustness of a phosphorescent metal complex is a crucial issue because the presence of electron(s) in the metal–ligand anti-bonding d_σ_ orbitals (for d^6^ and d^8^ electronic configuration), either through photoexcitation or thermal population, will decrease the strength of the M–L bond, resulting in its rupture. It is well-documented that the photosubstitution/photodissociation of [Ru(bpy)_3_]^2+^ (bpy = 2,2′-bipyridine) in solution occurs *via* thermally populated metal-centred (MC) d–d states.^28,29^ The rupture of Ir–L bonds *via* strongly structurally distorted d–d states has been reported to account for the degradation of Ir(iii) dopants in OLEDs.^26^

In this perspective, we describe the factors that affect the non-radiative decay rate and stability of luminescent Pt(ii) complexes, followed by a discussion of the recent advances made in phosphorescent Pt(ii) complexes which display high emission efficiency and/or high stability. In particular, the latest reports on luminescent Pt(ii) complexes supported by tetradentate ligands are summarized. Based on experimental findings and theoretical calculations, we try to correlate superior emission properties and robustness with structure in selected classes of phosphorescent Pt(ii) complexes. In addition to being used as phosphorescent OLED dopant materials, the self-assembly and bioimaging applications of luminescent Pt(ii) complexes are highlighted.

## 2 Design principles for highly robust, strongly emissive Pt(ii) complexes

### 2.1 Factors affecting phosphorescence quantum yield

The phosphorescence quantum yield (*φ*_P_) of the triplet excited state is determined by the radiative (*k*_r_) and non-radiative (*k*_nr_) decay rates:
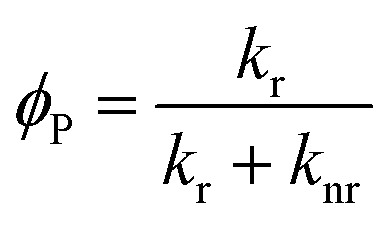


Among all the variables, suppressing the non-radiative decay rate *k*_nr_ is instrumental to achieving efficient phosphorescence. To this end, two main approaches have been employed: (1) decreasing the excited state structural distortion, and (2) altering the coordination environment so as to destabilize the metal-centred (MC) ligand-field excited states.

#### 2.1.1 Minimizing excited state structural distortion

**Fig. 2:**
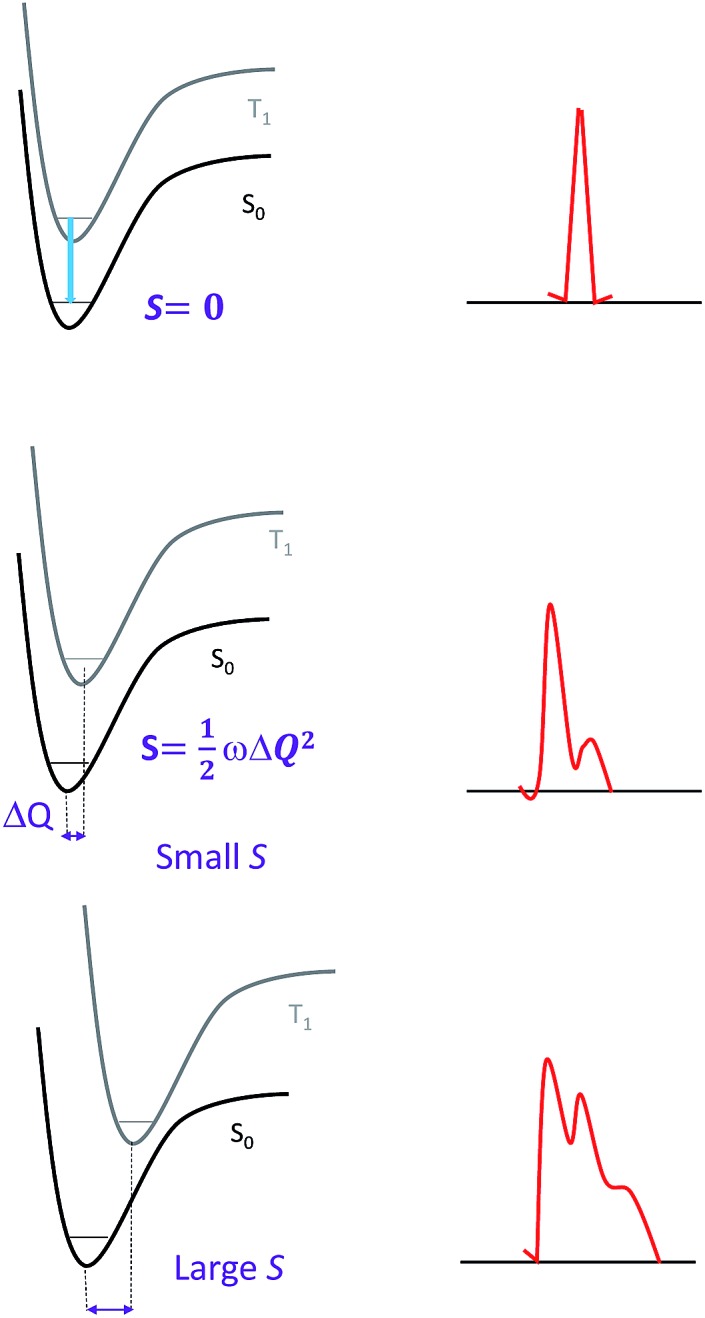
Correlations between the structural distortion of the triplet excited state with respect to the ground state and the emission spectrum: (top) sharp line emission with no structural distortion (*S* = 0); (middle) narrow bandwidth spectrum with a small structural distortion (*i.e.*, *S* is small); and (bottom) broad bandwidth spectrum yet with the vibrational signature of a larger structural distortion (*i.e.*, *S* is large); for an even larger *S*, only a broadband spectrum is observed.

Unlike d^6^ Ru(ii) and Ir(iii) complexes that have an octahedral coordination environment, Pt(ii) complexes usually adopt a square planar coordination geometry. The vacant axial coordination site(s) associated with square planar coordination geometry renders the excited state more flexible in undergoing structural reorganization, thus facilitating non-radiative decay. In addition, while the emitting triplet excited states of Ru(ii) and Ir(iii) complexes are usually ^3^MLCT in nature (MLCT = metal-to-ligand charge transfer), the emissions of Pt(ii) complexes usually have a ^3^MLCT character mixed with a significant ligand character, for instance, from intraligand charge transfer (^3^ILCT) and ligand-centred ^3^π–π* excited states. Therefore, one possible avenue to diminish *k*_nr_ is to design ligands for which the excited state structural distortion is small. Specifically, the so-called Huang–Rhys factor *S* serves to quantify the structural distortion Δ*Q* of the excited state with respect to the ground state. If *S* = 0, the excited state and the ground state have the same equilibrium geometries and only a sharp peak corresponding to a 0–0 transition is observed (Fig. 2). With an increase in Δ*Q* and hence an increase in *S*, a vibronic progression is observed (Fig. 2); the relative intensity of the 0–0 line and the first vibronic peak 1–0 are given by *S*:
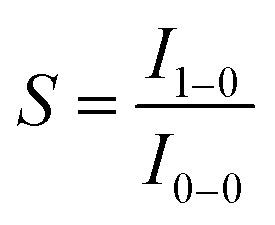


Since the overall emission bandwidth is affected by the intensity of all the vibronic progressions, a smaller *S* also implies a spectrum of narrow bandwidth and higher colour purity. Thus, complexes with highly rigid scaffolds are advantageous for developing luminescent materials with high emission quantum efficiencies.

**Fig. 3:**
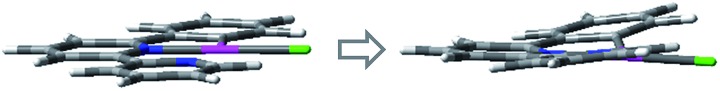
Optimized geometries of the ground state S_0_ (left) and the lowest triplet excited state T_1_ (right) of [Pt(C^N^N)Cl] **4** (adapted with permission from ref. 31. Copyright 2009, Wiley-VCH Verlag GmbH & Co. KGaA).

Spectroscopic and computational studies have revealed that the non-radiative decay rate of luminescent pincer-type cyclometalated Pt(ii) complexes can be profoundly affected by the ligands coordinated to Pt(ii). As depicted in Fig. 3, there is usually a significant structural distortion from a coplanar ground state geometry to a bent conformation in the T_1_ excited state for the cyclometalated Pt(ii) complexes that are non- or weakly emissive.^30–32^ Computational studies revealed that the quasi-degeneracy of the HOMO and H–1 orbitals and steric strain between the cyclometalated and ancillary ligands contribute to this type of excited state structural distortion that leads to very fast non-radiative decay.^31,32^ It has been pointed out that, in the design of efficient phosphorescent Pt(ii) emitters, the relative dispositions of coordinating donor atoms to the Pt(ii) ion and the site of π-conjugation should be taken into consideration.^31,32^

#### 2.1.2 Altering ligand-field splitting

Another tactic to decrease *k*_nr_ is pushing the MC ^3^d–d excited states well above the emitting triplet excited state. The thermal population of the strongly anti-bonding 5d_*x* ^2^–*y* ^2^_ orbital decreases the Pt–L bonding character, thereby providing an efficient non-radiative decay channel *via* severe structural distortions in the excited state. A common approach is to use strong σ-donor ligands, such as C-deprotonated cyclometalated ligands, N-heterocyclic carbenes (NHCs), and phenoxide ions.

### 2.2 Factors affecting the stability of phosphorescent metal complexes

Degradation of phosphorescent transition metal complexes usually occurs *via* M–L bond rupture. The tactics to reduce the propensity of M–L bond dissociation lie in (1) strengthening M–L bonds using strong σ-donor ligands and (2) using multidentate ligands. In general, transition metal complexes supported by multidentate ligands are more stable than those with monodentate ligands having comparable donor strength (termed the *chelate effect*); this is the result of the entropy effect in chelation reactions.^33^ In addition, the stability of transition metal complexes is influenced by the chelate ring size: 6-membered rings confer higher stability than 5-membered ones.^33^

### 2.3 Multidentate ligands for high efficiency and stability

**Scheme 1:**
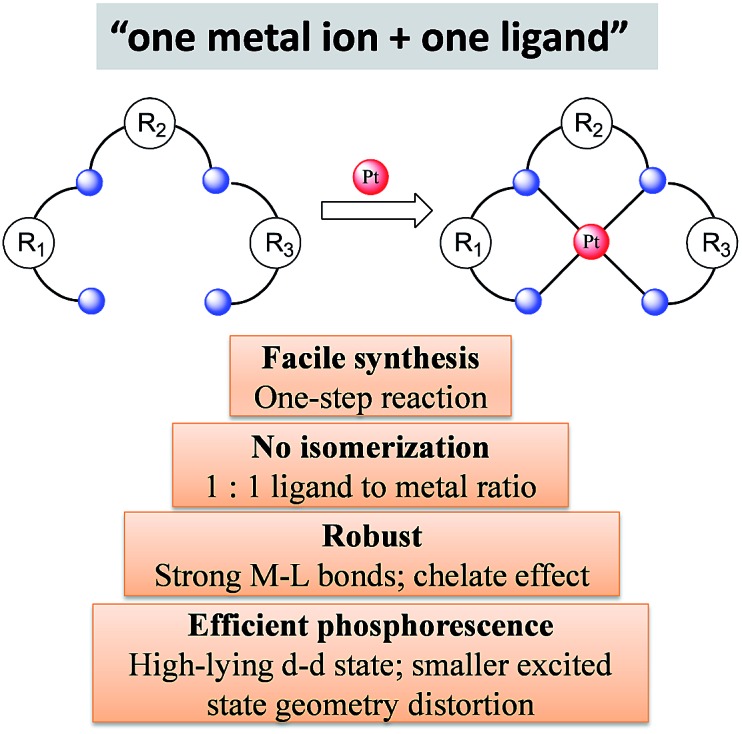
Illustration of the “one metal ion + one ligand” approach.

Considering the structural factors that affect emission efficiency, colour purity, and robustness, rigid multidentate ligand scaffolds containing strong σ-donor atoms are beneficial for the development of highly robust phosphorescent Pt(ii) complexes (Scheme 1). This design principle is reminiscent of the Ru(ii) polypyridyl complexes supported by “cage” or “hemicage” ligands, which have been shown to display superior emission properties and high photostability against light-induced substitution and decomposition reactions.^28,29,34,35^ In the literature, there are few examples of the “one metal ion + one ligand” approach to develop luminescent d^6^ Ir(iii) complexes.^36^ This may be due to the formidable challenges in synthesizing chemically inert, rigid hexadentate ligand scaffolds. In this regard, Pt(ii) complexes are advantageous over d^6^ octahedral metal complexes because tetradentate ligand scaffolds are relatively easy to construct and modify (Scheme 1). Indeed, the recently developed Pt(ii) complexes supported by tetradentate ligands have shown impressive emission efficiencies (*φ*_P_ ∼ 1) and exceptional thermal stability (*T*_d_ > 400 °C).^37^

## 3 Advances in luminescent Pt(ii) complexes

In this section, we describe the structures and emission properties of various types of Pt(ii) complexes which display high emission efficiency and/or high stability. Their pertinent photophysical data are summarized in Tables 1 and 2. Additionally, the performances of OLEDs doped with selected examples of these Pt(ii) emitters are discussed.

**Table 1:** Physical data of the Pt(ii) complexes containing bidentate or terdentate cyclometalated ligands

Coordination mode	Complex	Medium	*λ*_max_/nm	*φ*	*τ*/μs	*k*_r_/10^4^ s^–1^	*k*_nr_/10^4^ s^–1^	*T*_d_/°C	Ref.
[Pt(C^N)_2_]	**1**	THF	582	0.24	5.5	4.4	13.8	—^*c*^	40
[Pt(C^N)(L^X)]	**2**	2-MeTHF	485	0.22	4.5	4.9	17.3	—^*c*^	41
	**3**	CH_2_Cl_2_	595	0.47	2.6	18	21	—^*c*^	43
[Pt(C^N^N)X]	**4**	CH_2_Cl_2_	565	0.025	0.51	4.9	191	—^*c*^	44
	**5**	CH_2_Cl_2_	533, 569 (sh)	0.68	7	9.7	4.6	532	46
	**6**	CH_2_Cl_2_	521, 556 (sh)	0.99	5.5	18	—^*b*^	—^*c*^	32
	**7**	CH_2_Cl_2_	588, 632, 687 (sh)	0.25	10.1	2.5	7.5	—^*c*^	32
	**8**	CH_2_Cl_2_	495, 531	0.56	9.2	6.1	4.8	—^*c*^	50
[Pt(N^C^N)X]	**9**	CH_2_Cl_2_	491, 524, 562	0.6	7.2^*a*^	8.3	5.6	—^*c*^	51
	**10**	CH_2_Cl_2_	518, 548	0.37	5.0^*a*^	7.4	12.6	—^*c*^	54
	**11**	CH_2_Cl_2_	496	0.6	8.6^*a*^	7.0	4.7	—	55
[Pt(C^N^C)L]	**12**	CH_2_Cl_2_	589	0.26	15.8	1.6	4.7	343	65
	**13**	2-MeTHF	511	0.82	42	2.0	0.43	—^*c*^	30

^*a*^The lifetime *τ*_0_ at infinite dilution determined from the linear variation of the observed emission decay rate constant, *k*_obs_, as a function of the concentration of the complex.

^*b*^Value too low to be reported.

^*c*^Value not available from the literature.

### 3.1 Luminescent Pt(ii) complexes containing bidentate ligands

Homoleptic bis-cyclometalated Pt(ii) complexes were reported as early as 1984 by von Zelewsky *et al.* ^38–40^ This type of complex is known for its photochemical reactivity towards halogenated hydrocarbons. For example, the strongly orange light-emitting Pt(thpy)_2_ (**1**) (*φ* = 0.24 in THF) underwent an oxidative addition reaction with halogen-containing molecules, affording the corresponding Pt(iv) complexes which show blue-shifted phosphorescence.^40^ In view of the harsh conditions required for their preparation and their intriguing photolability, the phosphorescent bis-cyclometalated Pt(ii) complexes have been sparsely used as robust emitters. Alternatively, a number of heteroleptic cyclometalated Pt(ii) complexes with the formula [Pt(C^N)(acac)] (C^N denotes C-deprotonated 2-phenylpyridyl ligands and their derivatives; acac denotes deprotonated acetylacetone) have been reported and show intense phosphorescence in solution at room temperature.^41,42^ For example, **2** is strongly emissive with *λ*_max_ = 485 nm (*φ* = 0.22) in 2-methyltetrahydrofuran (2-MeTHF).^41^ It was reported that modification of the substitution on the cyclometalating C^N ligand could tune the emission energy to cover the visible spectral region.
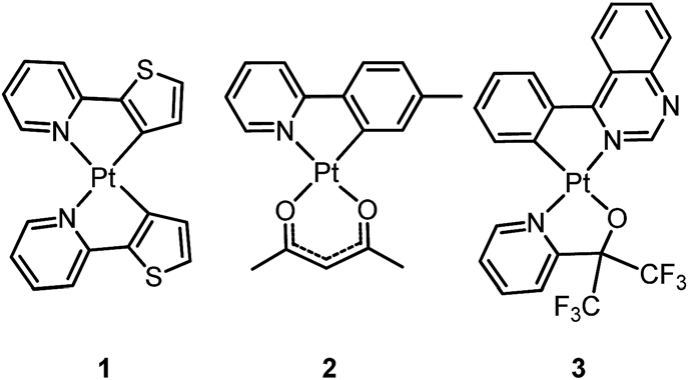


In the literature, heteroleptic cyclometalated Pt(ii) and Ir(iii) complexes, *i.e.*, [Pt(C^N)(L^X)] and [Ir(C^N)_2_(L^X)], have usually been used in the design of high performance triplet emitters.^5,7^ These complexes could be obtained from chloro-bridged [Pt(C^N)(μ-Cl)]_2_ or [Ir(C^N)_2_(μ-Cl)]_2_ dinuclear intermediates. Hence, modification of the cyclometalated and ancillary L^X moieties could be performed to systematically tune the physical and photophysical properties of these metal emitters. However, unlike the extensive reports on [Ir(C^N)_2_(L^X)] with L^X covering diverse mono-anionic bidentate ligands,^5,7^ only deprotonated acetylacetone (acac) and its derivatives are utilized as L^X ligands in the development of luminescent Pt(ii) complexes of the type [Pt(C^N)(L^X)].^42^ At this juncture, it should be noted that complex **3**, bearing 2-pyridyl hexafluoro-propoxide as the ancillary ligand, was reported to exhibit a high emission quantum yield of 0.47 (*λ*_max_ = 595 nm) in CH_2_Cl_2_.^43^ This finding highlights the promising prospect that phosphorescent [Pt(C^N)(L^X)] complexes have and indicates that they deserve further attention.

### 3.2 Luminescent Pt(ii) complexes containing a terdentate C-deprotonated ligand

**Scheme 2:**
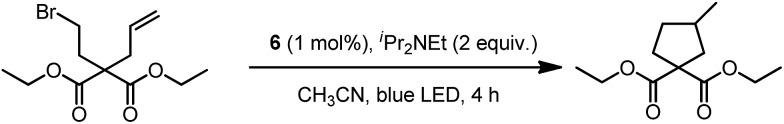


In 1999, Che and co-workers described luminescent pincer-type Pt(ii) complexes supported by C-deprotonated 6-aryl-2,2′-bipyridine ligands.^44^ Compared to [Pt(trpy)Cl]^+^ (trpy = 2,2′:6′,2′′-terpyridine), which is non-emissive under ambient conditions, complex **4** is emissive in CH_2_Cl_2_ (*φ* = 0.025) at room temperature, with the lowest triplet excited state having a ^3^MLCT character. It was suggested that the covalent Pt–C bond is the root cause for the enhanced emission properties of this complex in solution. Modification of either the C^N^N moiety or ancillary ligand has led to a number of luminescent Pt(ii) complexes with improved emission quantum yields and enhanced stability.^45,46^ For example, by extending π-conjugation of the C-deprotonated (C^N^N) moiety, strongly luminescent **5** (*λ*_max_ = 533 nm and *φ* = 0.68 in CH_2_Cl_2_) was prepared.^46^ Computational studies revealed that the extended π-conjugation of the cyclometalated ligand in **5** removes the quasi-degeneracy of the HOMO and H–1 orbitals, and hence the structural distortion between the T_1_ excited state and ground state is smaller when compared to that of **4**. This accounts for the low *k*_nr_ value of **5** in comparison to **4** (*k*_nr_ = 1.9 × 10^6^ s^–1^ for **4** and 4.6 × 10^4^ s^–1^ for **5**).^31^ It is of note that, because of the rigid π-conjugated terdentate cyclometalated ligand, **5** has a high thermal stability with a decomposition temperature (*T*_d_) of 532 °C in N_2_. Using **5** as a light-emitting material, a high-performance yellowish-green OLED was fabricated with a maximum brightness of 63 000 cd m^–2^. Che and co-workers also reported an efficient white OLED (WOLED) by combining emissions from a blue fluorescent material and a green-yellow emitting analogue of **5** in which the ^*t*^Bu groups were changed into CF_3_.^47^ The device having dual emitting layers exhibited a balanced white-light with a CIE of (0.30, 0.32). The maximum EQE reached 11.8%. Very recently, Che and co-workers reported that **6** (*λ*_max_ = 521 nm) displays a phosphorescence efficiency of almost one in room temperature solution.^32^ Computational studies revealed that **6** shows the smallest structural distortion from S_0_ to T_1_ along the high-frequency normal modes of the complexes studied therein. The excited state geometry distortion was found to be highly dependent on the site of π-conjugation and nature of the additional ancillary ligand. A yellowish-green OLED doped with **6** showed a maximum EQE of 22.8%, which is among the highest values ever reported for OLEDs with Pt(ii) dopants. With a thiophene-containing π-conjugated ligand, complex **7** has an emission with *λ*_max_ = 588 nm in CH_2_Cl_2_.^32^ An efficient red-emitting OLED fabricated using **7** as the dopant showed a maximum EQE of 22.1%, which is comparable to the best values of red-emitting OLEDs based on Ir(iii) complexes.^48^ The pincer-type cyclometalated Pt(ii) complexes have been employed as photosensitizers or photocatalysts for solar energy conversions. Fu and co-workers showed that [Pt(C^N^NPhMe)Cl] (HC^N^NPhMe = 4-(*p*-tolyl)-6-phenyl-2,2′-bipyridine) was an effective photosensitizer for hydrogen evolution from water and afforded a much higher turnover number than its terpyridyl analogue under the same experimental conditions.^49^ Complex **6** has been examined by Che as a photocatalyst for visible light-induced reductive C–C bond formation reactions (Scheme 2).^32^ With diisopropylethylamine (^i^Pr_2_NEt) as a sacrificial electron donor in CH_3_CN, and after 4–8 hours of irradiation using a blue LED, a series of alkyl bromides underwent intramolecular C–C bond formation with conversions and yields of up to 99% and 78%, respectively. This complex also catalysed the light-induced intermolecular C–C bond formation from benzyl chloride.
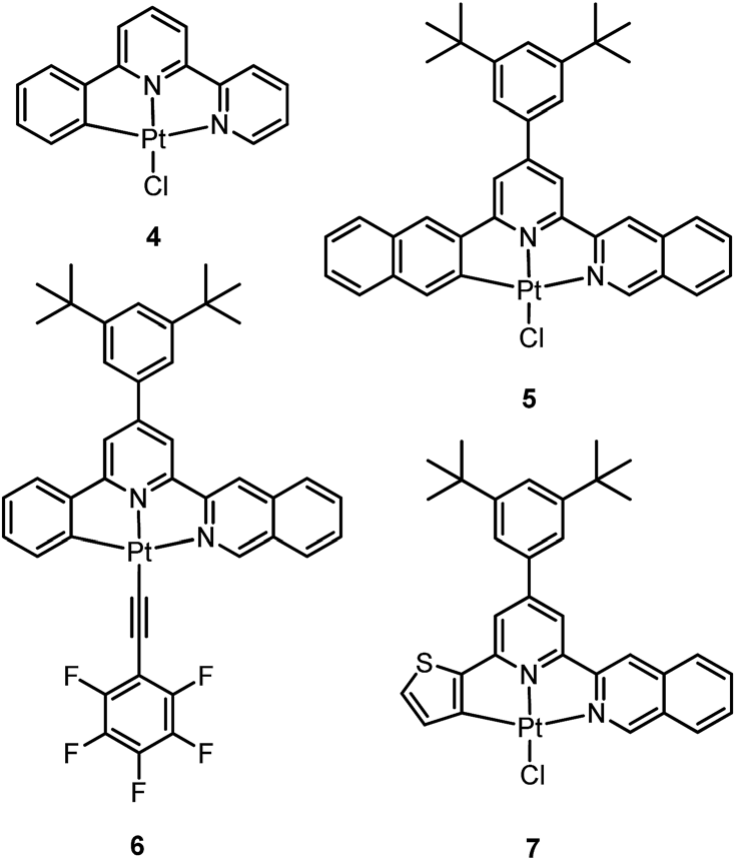


Huo and co-workers reported the structural modification of a terdentate C-deprotonated cyclometalated ligand resulting in the change of its [Pt(C^N^N)] motif from having a fused 5,5-membered metallacycle (as in **4–7**) to having a 5,6-membered one (as in **8**).^50^ Complex **8** exhibits substantially improved phosphorescence (*φ* = 0.56 and *τ* = 9.2 μs in CH_2_Cl_2_) compared to its fused 5,5 analogue, [Pt(C^N^N)(C≡CPh)] (*φ* = 0.04 and *τ* = 0.4 μs in CH_2_Cl_2_) (Chart S1, ESI†).^45^ The substantial difference in the *k*_nr_ values of both complexes (*k*_nr_ = 4.8 × 10^4^ s^–1^ for **8** *vs.* 2.4 × 10^6^ s^–1^ for [Pt(C^N^N)(C≡CPh)]) reveals that the phosphorescence efficiency is strongly affected by the non-radiative decay rate constant. On the one hand, the slower non-radiative decay rate of **8** is probably due to the stronger donor strength of **8**,^50^ while on the other hand, computational studies have shown that the emitting triplet excited state of [Pt(C^N^N)(C≡CPh)] is a ^3^[π(C≡CPh) → π*(C^N^N)] ligand-to-ligand charge transfer (LLCT) mixed with ^3^[dπ(Pt) → π*(C^N^N)] (MLCT), and that of **8** is ^3^π–π*(C^N) mixed with ^3^[dπ(Pt) → π*(C^N)] (Fig. S1, ESI†). These calculations are consistent with the experimental finding that [Pt(C^N^N)(C≡CPh)] and **8** display structureless and vibronic-structured emission profiles, respectively. As the emissive excited state of [Pt(C^N^N)(C≡CPh)] involves distortion of the C≡C bond while that of **8** does not, the presence of an additional effective high-frequency accepting mode, *ω*_C≡C_, would lend the former complex a faster non-radiative decay rate (see details in the ESI†). This difference in the nature of the T_1_ excited state is attributed to the presence of an additional amine bridge in **8** that causes the C^N moiety and pyridyl ring to be non-planar to each other, while the C-deprotonated C^N^N cyclometalated ligand has a relatively planar geometry in the case of [Pt(C^N^N)(C≡CPh)] (Fig. S2, ESI†). Moreover, as stated by the energy-gap law, the lower emission energy of [Pt(C^N^N)(C≡CPh)] may also contribute to the observed faster non-radiative decay rate.
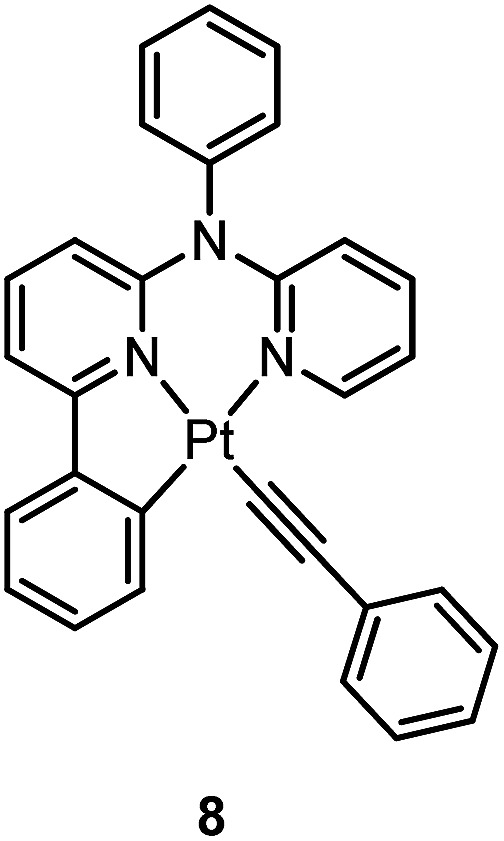


Williams and co-workers described Pt(ii) complexes (**9** and its derivatives) supported by terdentate cyclometalated ligands derived from C-deprotonated 1,3-bis(2-pyridyl)benzene (N^C^N) ligands.^51^ A high emission efficiency of 0.6 was recorded for **9** in CH_2_Cl_2_ and the emitting triplet excited state has been characterized to be metal-perturbed ^3^IL (IL = intraligand) in nature. Compared to [Pt(C^N^N)Cl] (**4**), shorter Pt–C bonds were observed for **9** and its derivatives.^51,52^ This may result in **9** having a higher-lying d–d excited state. The finding of the emission lifetime to be insensitive to temperature rules out the possibility of a non-radiative decay pathway occurring *via* a d–d state.^53^ The [Pt(N^C^N)Cl] complexes show a strong tendency to undergo excimer formation at elevated concentrations in solution, and aggregation and/or excimer formation in the solid state. Taking advantage of this feature, modification of either the cyclometalating moiety or variation of the fourth auxiliary ligand can tune the energy of frontier molecular orbitals and/or perturb the extent of intermolecular interactions, leading to a shift in the emission to the near-infrared (NIR) region. For instance, attaching electron-withdrawing –CF_3_ groups stabilizes the pyridyl-localized LUMO of **10** and hence its excimeric emission occurs at a low energy of 756 nm in CH_2_Cl_2_.^54^ Coordination of SCN^–^ to Pt(ii), as in the case of **11**, does not affect the excited state energy in comparison to its chloride counterpart, but alters the solid-state packing, resulting in a shorter Pt···Pt contact of 3.3 Å. As a consequence, an OLED based on a neat-film of **11** shows EL at 855 nm.^55^ In addition to the promising NIR emission due to aggregation or excimer formation, cyclometalated pincer-type Pt(ii) complexes are also promising candidate materials for single-dopant WOLEDs through a combination of both their monomeric blue-to-green emission and excimeric red emission (*vide infra*).^56–58^ 
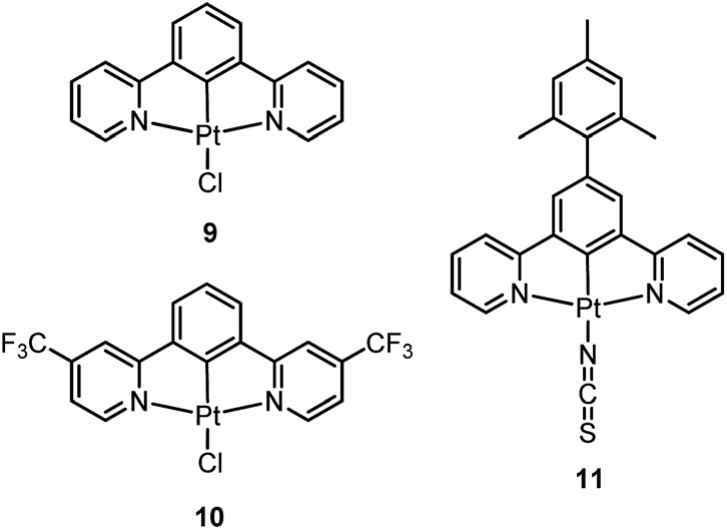


Pt(ii) complexes supported by doubly C-deprotonated 2,6-diphenylpyridine (C^N^C) ligands constitute another class of cyclometalated Pt(ii) complexes which possess rich photophysical properties.^59–64^ Despite the presence of two covalent Pt–C bonds, most of the [Pt(C^N^C)L] type complexes are non-emissive or weakly emissive in solution at room temperature. Computational studies revealed that these complexes undergo significant structural distortions upon shifting from S_0_ to T_1_ state.^31^ In 2012, Che and co-workers reported the first examples of organoplatinum(ii) complexes bearing functionalized (C^N^C) ligands that are emissive in solution at room temperature (*e.g.*, *φ* = 0.26 for **12**).^65^ The emission switch-on property is achieved by extending the π-conjugation of the (C^N^C) ligand, thereby shifting the lowest electronic excited states to be predominantly ^3^IL in nature. Because of the more rigid ligand framework, the excited state structural distortion of the new emitters is greatly reduced. High thermal stability (*T*_d_ > 300 °C) renders **12** a suitable dopant for OLED applications; a red OLED with CIE coordinates of (0.65, 0.35) was fabricated and a maximum EQE of 12.6% was obtained. Very recently, Yersin *et al.* developed a brightly luminescent Pt(ii) complex **13** (*φ* = 0.82 in 2-MeTHF) which is supported by a bulky and rigid carboranyl-phenylpyridine ligand.^30^ Photophysical studies revealed an exceptionally small non-radiative decay rate constant that is in the order of 10^3^ s^–1^. DFT calculations showed that no bent conformation, which is commonly encountered in other non-emissive [Pt(C^N^C)X] (X = auxiliary ligand) complexes, can be observed in the T_1_ excited state of **13**, thereby significantly decreasing the non-radiative decay rate.
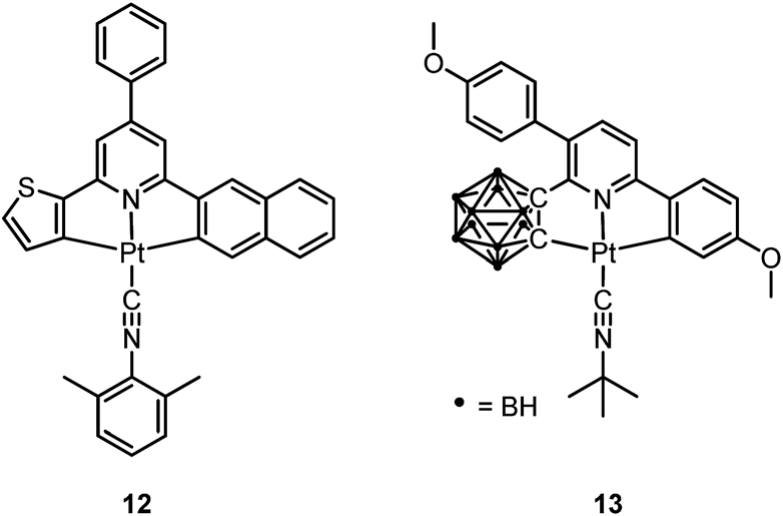


### 3.3 Luminescent Pt(ii) complexes supported by a tetradentate ligand

Pt(ii) porphyrin complexes are well-known to be robust phosphorescent emitters as a result of the rigid macrocyclic porphyrin ligand scaffold. With an extended π-conjugated porphyrin ligand, these complexes display red or NIR ^3^IL emission with very long lifetimes.^66–69^ In recent years, there has been a surge of interest to develop luminescent Pt(ii) complexes supported by non-porphyrin tetradentate ligands. By judiciously designing the tetradentate ligand, the emission energy can be tuned throughout the entire visible spectral region. This type of Pt(ii) complex usually has high thermal stability and the emissive excited states show a relatively slow non-radiative decay rate.
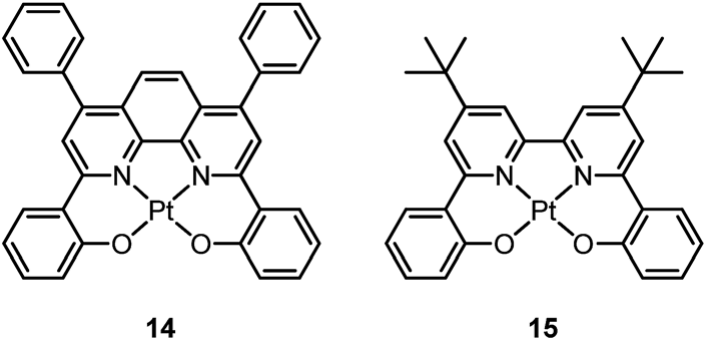


**Fig. 4:**
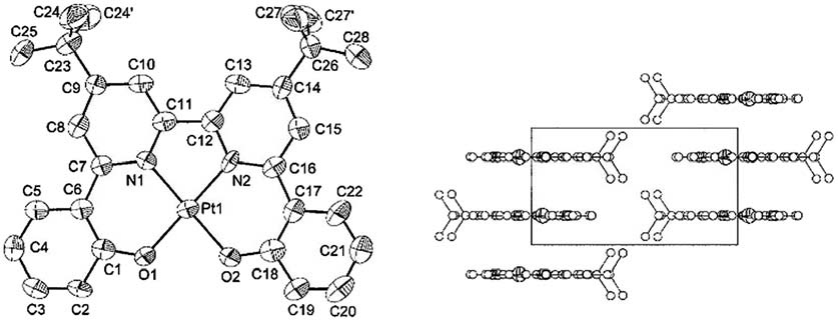
Perspective view (50% thermal probability) (left) and packing arrangement along the *ab* plane (right) of **15** (adapted with permission from ref. 70. Copyright 2003, Wiley-VCH Verlag GmbH & Co. KGaA).

In 2003, Che and co-workers reported the first series of phosphorescent Pt(ii) complexes supported by tetradentate dianionic bis(phenoxy)diimine (N_2_O_2_) ligands (**14** and **15**) that have fused 6,5,6-membered rings.^70^ The Pt(ii) ions in the crystal structures of both **14** and **15** adopt a planar coordination geometry and the two Pt(N_2_O_2_) frameworks are highly planar (Fig. 4). Complexes **14** and **15** are stable up to 440 and 530 °C, respectively, in N_2_. Notably, they also show very high thermal stability in air with significant weight loss at temperatures above approximately 380 °C. Complexes **14** and **15** display structureless emissions in CH_2_Cl_2_ at room temperature with *λ*_max_ = 586 nm (*φ* = 0.6; *τ* = 5.3 μs) and 595 nm (*φ* = 0.1; *τ* = 1.9 μs), respectively. Their potentials as phosphorescent OLED emitters were examined. The maximum brightness (9330 cd cm^–2^) and power efficiency (1.44 lm W^–1^) were recorded for a device using **15** as the dopant. Despite the much higher emission quantum yield of **14** in CH_2_Cl_2_, the device doped with **14** exhibited a low performance that was ascribed to intermolecular quenching (including self-quenching). In contrast, the bulky *tert*-butyl groups in **15** are believed to reduce the intermolecular interactions.

**Fig. 5:**
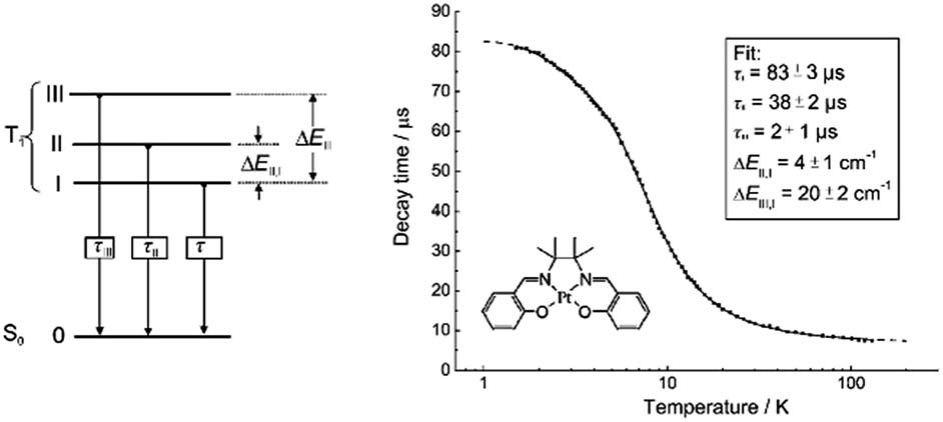
Schematic energy-state diagram for the substates of the emitting T_1_ state of a transition metal complex (left). Plot of emission decay times of **16** in THF (∼10^–5^ M) *versus* temperature and the fit to the equation describing the temperature dependence of the measured decay time in a model of three substates (right) (adapted with permission from ref. 72. Copyright 2010, Wiley-VCH Verlag GmbH & Co. KGaA).

**Fig. 6:**
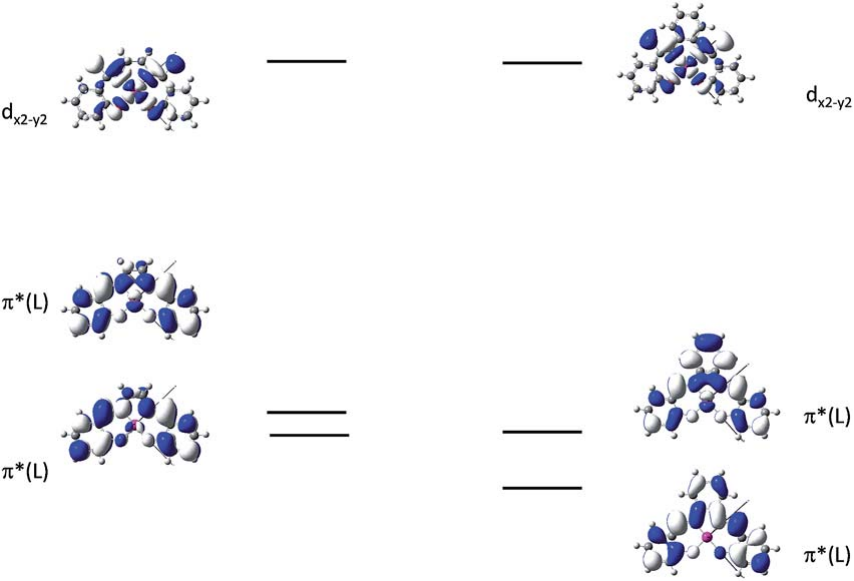
MO diagrams of **16** and **17** showing the ^3^MLCT–^3^d–d energy gap change upon ligand modification (adapted with permission from ref. 73. Copyright 2014, Wiley-VCH Verlag GmbH & Co. KGaA).

In 2004, Che and co-workers reported another tetradentate N_2_O_2_ ligand system, *i.e.*, Schiff base, that can be used for the preparation of phosphorescent Pt(ii) emitters.^71^ Detailed photophysical studies of a series of Pt(ii) Schiff base complexes were later presented in 2010.^72^ These types of complex are attractive in the context of practical applications because of the straightforward preparation of the Schiff base ligands and the simplicity of scaling up for mass production. It is also well-known that Schiff bases readily form stable transition metal complexes. Not surprisingly, all the Pt(ii) Schiff base complexes are stable in the solid state, and no decomposition or ligand dissociation was observed in EtOH, 2-propanol, DMSO, or CH_3_CN under ambient conditions for one month. The Pt(ii) Schiff base complexes display excellent thermal stability with *T*_d_ of up to 495 °C in N_2_.^72^ Complex **16** shows an emission with *λ*_max_ = 546 nm in benzene. This complex exhibits the highest reported emission quantum yield (*φ* = 0.27) among this class of Pt(ii) emitters. Structural diversity of the Schiff base ligand allows facile tailoring of both the physical and photophysical properties of this class of complexes. Replacing the ethylene linker with a phenyl ring resulted in a red-shifted emission as in the case of **17** (*λ*_max_ = 628 nm and *φ* = 0.26 in benzene). Zero-field splitting (ZFS) can be used to estimate the extent of MLCT perturbation and SOC efficiency of the emitting triplet state as well as to assess the suitability of an emitter for OLED fabrication.^1,2^ The value of ZFS of selected complexes was determined by measuring their temperature-dependent lifetimes in the range of 1.5–130 K. As shown in Fig. 5, the total ZFS for **16** was estimated to be 20 cm^–1^, belonging to the intermediate range in a library of triplet emitters examined by Yersin and co-workers.^72^ The emitting triplet state was assigned to have ILCT character with significant MLCT perturbation. DFT/TDDFT studies also supported this assignment. Che and co-workers have extended the theoretical studies to the substituent effects on the luminescence efficiency of the Pt(ii) Schiff base complexes.^73^ Replacing the ethylene bridge (**16**) of the Schiff base ligand with a π-conjugated phenylene bridge (**17**) not only widens the ^3^MLCT–^3^d–d energy gap due to a lowered ligand π* orbital (relative to the d_*x* ^2^–*y* ^2^_ orbital) in **17** (Fig. 6), but also results in a smaller Huang–Rhys factor (*S*_M_ = 1.05 and 0.89 for **16** and **17** respectively). As such, though the emission energy of **17** is smaller than that of **16** and the former should have a faster non-radiative decay rate than the latter based on the energy gap law, both complexes have similar *k*_nr_ values in CH_3_CN at room temperature (*k*_nr_ = 2.31 × 10^5^ s^–1^ for **16** and 2.24 × 10^5^ s^–1^ for **17**)^72^ due to the smaller structural distortion in **17**. It was also demonstrated in the same work that the nature of the substituents (**17–19**) can modify the SOC and frequency shifts of the low-frequency modes between the T_1_ and S_0_ states. Inclusion of both factors in addition to the structural distortion allowed quantitative computation of the phosphorescence efficiency. An OLED doped with **16** at an optimized doping concentration of 4.0 wt% exhibited a maximum EQE, current efficiency, power efficiency, and brightness of 11%, 31 cd A^–1^, 14 lm W^–1^, and 23 000 cd m^–2^, respectively.^71^ Moreover, this device gave stable EL spectra over a wide range of operating voltages from 3 to 16 V. Using the same device configuration as that for **16**, a stable red-OLED using **17** as the dopant was achieved with a maximum EQE and power efficiency of 9.4% and 4.9 lm W^–1^, respectively. Notably, this device showed a lifetime of more than 20 000 h at 100 cd m^–2^ and the performance was further improved to an impressive 77 000 h at 500 cd m^–2^ by modifying the device configuration.^72^ However, due to the severe self-quenching effect (*k*_q_ = 6.9 × 10^8^ M^–1^ s^–1^), the optimal device can only be fabricated with a low doping level of **17** (<1.5 wt%). Recently, Che and co-workers prepared a series of derivatives of **17** bearing bulky groups, an example of which is **20**.^74^ Complex **20**, having norbornene moieties in the ligand scaffold, displays an emission quantum yield of 0.20 with *λ*_max_ = 624 nm in CH_2_Cl_2_ and a significantly reduced emission self-quenching rate constant (*k*_q_) of 1 × 10^7^ M^–1^ s^–1^. As a result, an efficient red OLED could be fabricated at a higher doping concentration of 4 wt% and displayed a delayed efficiency roll-off. Wong *et al.* also reported an asymmetric Pt(ii) Schiff base complex **21** with bulky *tert*-butyl and triphenylamino groups introduced at different sites to prevent aggregation or excimer formation.^75^ Using **21** as the emitter, an efficient yellow OLED (*λ*_max_ = 564 nm) was fabricated at a high doping concentration (8.0 wt% in TCTA). The EQE, current efficiency, power efficiency, and maximum brightness were 8.3%, 23 cd A^–1^, 17 lm W^–1^, and 11 106 cd m^–2^, respectively.
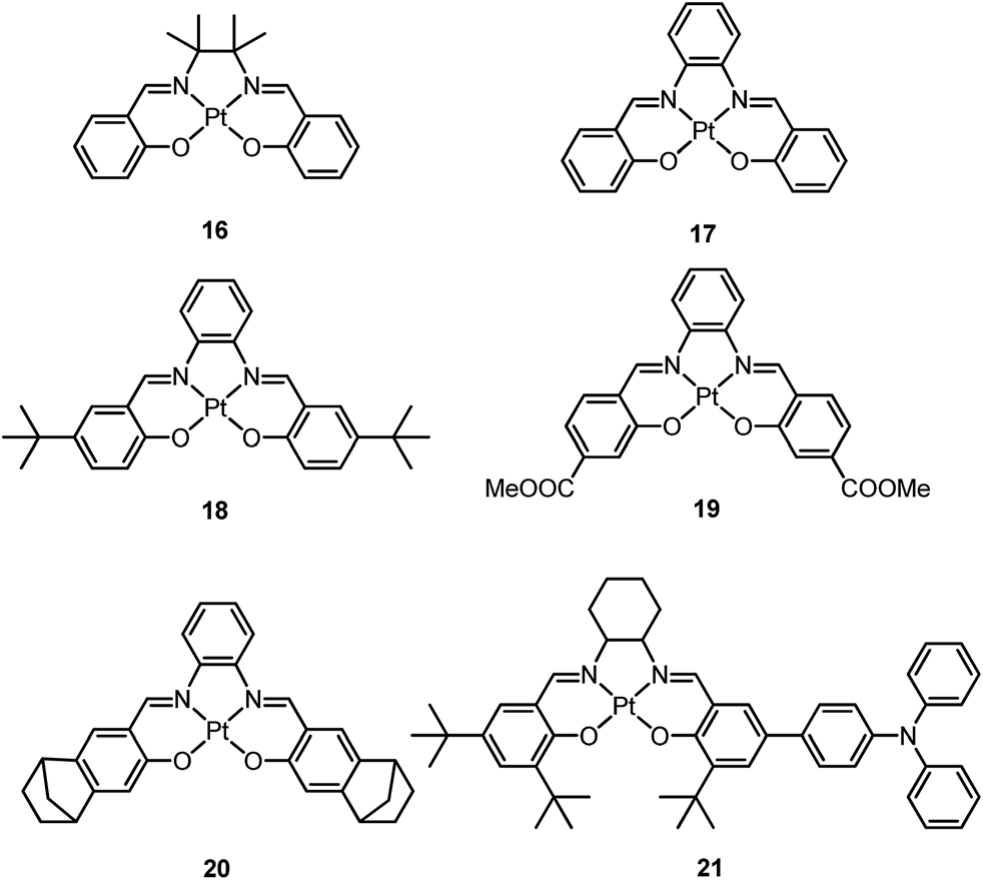


Che and co-workers previously developed a class of phosphorescent Pt(ii) complexes supported by tetradentate bis(pyrrole)-diimine (N_4_) ligands.^76^ Complex **22** displays a yellow emission in dilute CH_3_CN (concentration ∼ 1 × 10^–6^ M) with an emission quantum yield and lifetime of 0.097 and 4.2 μs, respectively. Interestingly, at elevated concentrations in either solution or CBP (CBP = 4,4’-bis(*N*-carbazolyl)-1,1’-biphenyl) film, **22** shows a red-shifted emission which has been attributed to the high propensity of **22** for excimer or oligomer formation. Thus, an OLED doped with **22** at 6.0 wt% afforded red electroluminescence with a peak maximum at 620 nm and CIE coordinates of (0.62, 0.38). The maximum EQE, current efficiency, power efficiency, and brightness for this device were 6.5%, 9.0 cd A^–1^, 4.0 lm W^–1^, and 11 100 cd m^–2^, respectively. High efficiencies were maintained even at a high brightness of 5000 cd m^–2^ (5.2%, 7.7 cd A^–1^, and 2.4 lm W^–1^). This work represents a proof-of-concept that excimer formation of Pt(ii) complexes can be used to achieve efficient low-energy emission. Indeed, NIR OLEDs based on emission from excimeric and aggregate species have been demonstrated by Williams on pincer-type Pt(ii) emitters.^54,77^ However, **22** exhibits a relatively low *T*_d_ of 288 °C in N_2_. This is attributed to the significant strain chelate present in **22** as this complex has a fused 5,5,5-membered metallacycle, without the optimal 6-membered rings. Very recently, Chi and co-workers reported a new type of highly emissive Pt(ii) complex supported by a tetradentate ligand with four N donor atoms.^78^ Complex **23** exhibits blue emission (*λ*_max_ = 461 nm, *φ* = 0.82) in CH_2_Cl_2_. This emission has been attributed to a ligand-centred π–π* transition. Complex **24**, having an acridine moiety, possesses a dominant charge-transfer character in its lowest triplet excited state. As a result, this complex displays significant solvatochromism. Upon switching the solvent from cyclohexane to CH_2_Cl_2_, the emission quantum yield and lifetime change from 0.13 and 20.1 μs to 0.88 and 2.9 μs, respectively. The increased *k*_r_ is due to the variation of emission character from π–π*/MLCT to ILCT/MLCT. Both **23** and **24** have been used for sky-blue OLED fabrications. The CIE coordinates, peak EQE, and maximum brightness were (0.190, 0.342)/(0.194, 0.391), 12.3%/15.3%, 1924/4121 cd m^–2^ for **23**/**24** based OLEDs.
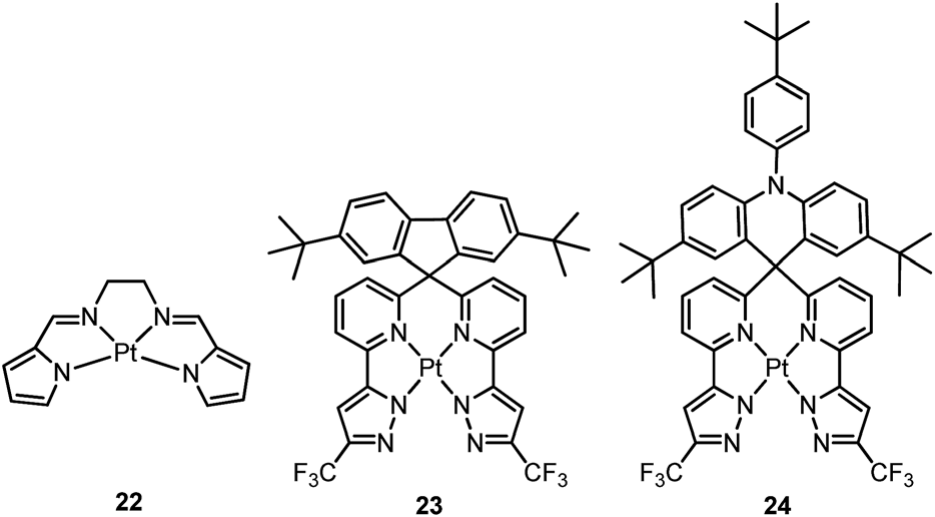


Recently, there has been considerable interest in tetradentate ligand systems containing deprotonated C-atoms and/or NHCs. These ligand systems have been shown to afford superior Pt(ii) emitters in terms of both emission efficiency and/or robustness. In 2009, Weck *et al.* developed complexes containing doubly cyclometalated tetradentate ligands (C^N^N^C).^79^ Complex **25** shows vibronic-structured phosphorescence in CH_2_Cl_2_ with a quantum yield of 0.58 (*λ*_max_ = 492 nm). In 2010, Huo and co-workers reported a related system in which the cyclometalated C^N motifs are bridged by an amino group as in the case of **26**.^80^ Complex **26** exhibits an intense green (*λ*_max_ = 512 nm) vibronic structured emission in 2-MeTHF with a quantum yield of 0.74. Upon incorporation of **26** at 4 wt% in a mixed host into an OLED, an excellent device performance with a maximum EQE of 14.7% was observed. Crystal structures reveal that the Pt–C distances of 2.00–2.01 Å for **25** and **26** are close to those of ∼2.00 Å for Pt(ppy)_2_ (ppy = deprotonated 2-phenylpyridine) while the Pt–N bond lengths of 2.05–2.08 Å are shorter than those of Pt(ppy)_2_ (∼2.13 Å). In the same work, Huo also reported the photophysical properties of the Pt(ii)–N^C^C^N complexes (*e.g.*, **27** and **28**) that are isomeric derivatives of the Pt(ii)–C^N^N^C ones.^80^ Similar to the findings for **25** and **26**, the formation of a metallacycle results in shorter Pt–C bond distances (∼1.96 Å) for **27**, when compared with those of *cis*-Pt(ppy)_2_ (∼1.99 Å). Complex **27** emits red light in 2-MeTHF with *λ*_max_ at 613 nm (*φ* = 0.14). Replacing the pyridyl ring in **27** with the less electron-accepting pyrazolyl ring resulted in a hypsochromic shift of the emission and enhanced emission efficiency, as in the case of **28** (*λ*_max_ = 486 nm, *φ* = 0.63). Compared to **27**, the enhancement of *φ* for **28** is attributed mainly to an increased *k*_r_ (*k*_r_ = 1.1 × 10^5^ s^–1^ for **28** *vs.* 1.8 × 10^4^ s^–1^ for **27**). However, DFT calculations show a comparable metal parentage in the HOMOs of **27** and **28** and, therefore, the metal character of the frontier orbital is not sufficient to account for such a large variation in the *k*_r_ value. In fact, the factors affecting the radiative decay rate constant are complex. It is necessary to take into account, in addition to spin–orbit coupling (SOC) matrix elements, the singlet–triplet energy gap and the oscillator strength of the transition from S_0_ to the singlet excited state from which the triplet excited state borrows intensity.^31^ Fukagawa and co-workers later modified **27** by attaching two *tert*-butyl groups to the *N*-pendant phenyl ring to give **29**, which emits red light (*λ*_max_ = 621 nm) with a quantum yield of 0.58 in a bis(benzo[*h*]quinolin-10-olato-*kN*,*kO*)beryllium(ii) (Bebq_2_) film.^81^ The optimized OLED doped with **29** showed good colour saturation with CIE coordinates of (0.66, 0.34), low driving voltage, high efficiency, and high operational stability. The maximum EQE of over 19% and maximum power efficiency of 30 lm W^–1^ were comparable to the highest values previously reported for red OLEDs using Ir(iii) complexes.^82–84^ The estimated half-life for the optimized OLED was about 10 000 h with an initial brightness of 1000 cd m^–2^.
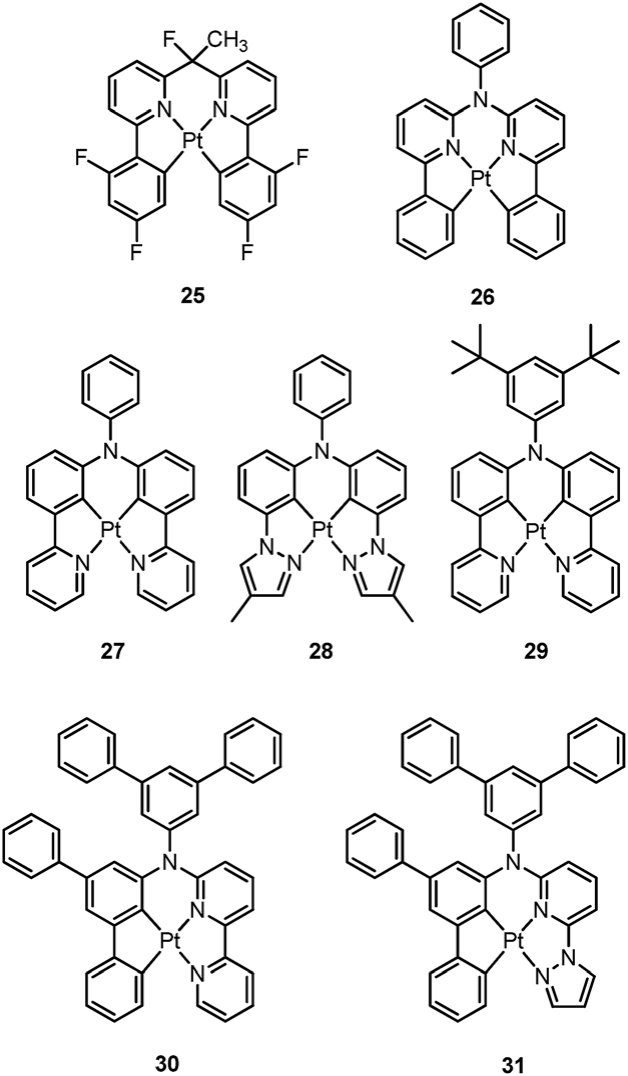


Huo and co-workers later reported the synthesis of Pt(ii)–C^C^N^N complexes **30** and **31**.^85^ Complex **30**, a closely-related analogue of **25** and **27**, is only weakly emissive (*φ* = 0.001) in the deep red region with a *λ*_max_ of 660 nm. Replacement of the pyridyl ring of **30** with a pyrazolyl ring results in bright yellow phosphorescence at *λ*_max_ = 550 nm (*φ* = 0.17). The Huang–Rhys factors for **30** and **31** were estimated to be 0.42 and 0.37 respectively, comparable to those found in Pt(ii)–C^N^N^C and Pt(ii)–N^C^C^N complexes, indicating that these complexes should have similar structural distortion between the emitting triplet excited state and ground state. The much faster non-radiative decay rate of **30** (*k*_nr_ = 1.2 × 10^6^ s^–1^) is therefore likely to be associated with its lower energy emission, a consequence of the “energy-gap law”.^86^ On the other hand, although DFT calculations show that the triplet excited states of **30** and **31** are ILCT mixed with MLCT, the distinctly larger *k*_r_ of 3.9 × 10^4^ s^–1^ for **31** over **30** (*k*_r_ = 1.2 × 10^3^ s^–1^) reflects the significant dependence of *k*_r_ on subtle ligand modification, again as for that observed for **28** over **27**.
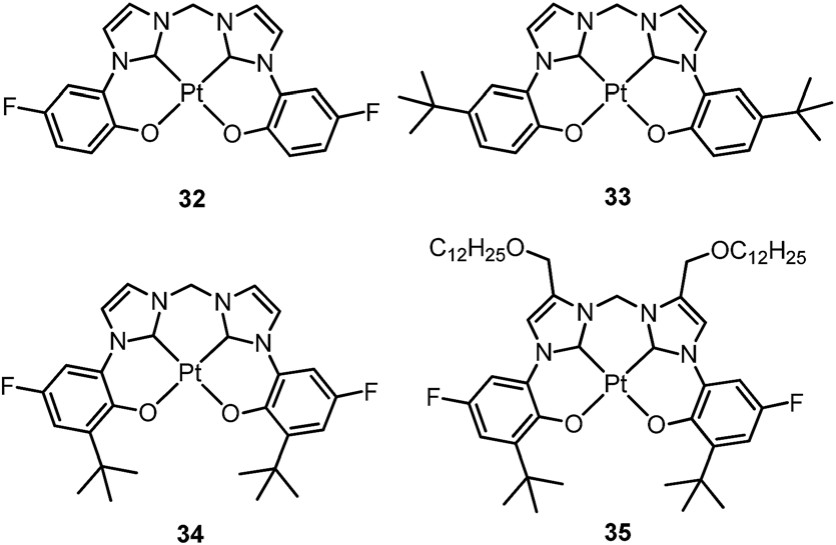


**Fig. 7:**
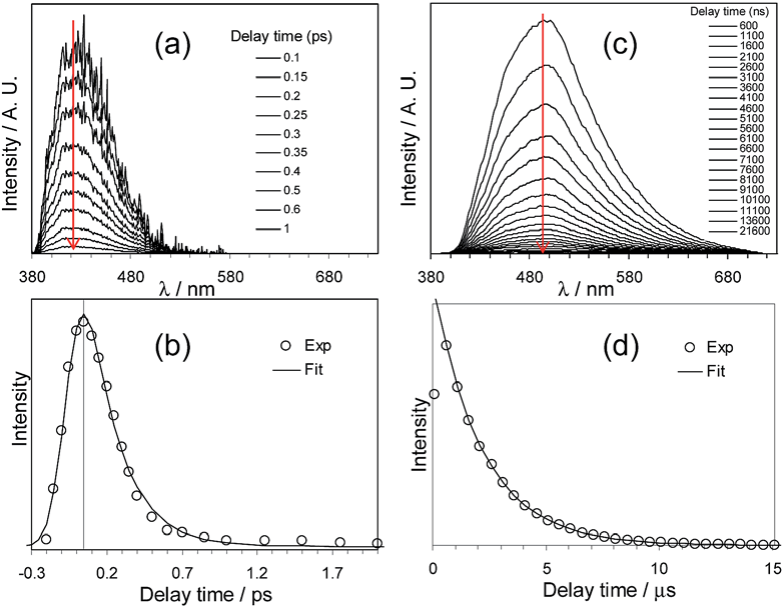
fs-TRF and ns-TRE spectra (a and c) and decay profiles (b and d) recorded at the various denoted delay times after 355 nm excitation of **33** in DMF (reproduced from ref. 89 with permission from the Royal Society of Chemistry).

Che's and Strassner's groups independently reported tetradentate Pt(ii) complexes containing dianionic bis(phenolate-NHC) ligands.^87,88^ The fused 6,6,6-membered metallacycle allows an ideal coordination geometry around the Pt atom with all bite angles being 90 ± 2°.^87^ As expected, the optimum chelate ring size together with the strong σ-donating C and O donor atoms render these complexes with good thermal stability with a *T*_d_ of up to ∼410 °C in N_2_. In poly(methyl methacrylate) (PMMA), both **32** and **33** exhibit deep blue phosphorescence (*λ*_max_ ∼ 450 nm) with emission efficiencies of *ca.* 0.3. This is the first report on deep blue emission from Pt(ii) complexes supported by tetradentate ligands.^87^ On the basis of photophysical studies and DFT calculations, the emitting states have been assigned to have mainly ILCT character with MLCT perturbation, similar to the Pt(ii) Schiff base complexes. The femtosecond spectroscopic technique has proven useful in providing valuable information to understand the ultrafast excited state dynamics. As depicted in Fig. 7, upon photoexcitation, the S_1_ state of **33** undergoes an ultrafast ISC process with a fluorescence lifetime of 0.19 ps, revealing that the involved MLCT character is sufficient for promoting the ISC process.^89^ Recently, Che and co-workers prepared two new complexes **34** and **35**, containing *para*-F and *ortho*-^*t*^Bu groups (Scheme S1 in ESI†). These complexes show improved emission quantum yields of 0.43 and 0.37 in THF, respectively (Fig. S3, ESI†). The *k*_r_ values (4.4–5.1 × 10^4^ s^–1^) of **32–35** are comparable and the emission variations mainly arising from their differences in non-radiative decay rates (Table 2). It is rational to assume that the ligand-field strength, and thus d–d energy level, is not much affected by the peripheral substitutions on the ligand in the present system. Given their close emission energies (443–465 nm), the non-radiative relaxations for **32–35** *via* thermal population of the ^3^d–d states are envisaged to be comparably marginal. For the same reason, the extent of the energy-gap law contribution to the non-radiative decay should be similar. Thus, excited state (T_1_) geometry distortions with respect to the ground state are conceived to be the cause for such differences in the non-radiative decay rate for **32–35**. For complexes having the same excited state nature (^3^LLCT/^3^MLCT), an indicator of the geometry distortion from S_0_ to T_1_ is the absorption–emission Stokes shift. A comparison of the absorption and emission spectra of **32–34** in DMF is shown in Fig. S4 (ESI†). The *para*-F and *ortho*-^*t*^Bu substituents are effective in reducing excited state structural distortions, leading to decreased absorption–emission Stokes shifts from **33** to **32** and **34**.

**Table 2:** Physical data of the Pt(ii) complexes supported by a tetradentate ligand

Coordination mode	Complex	Medium	*λ*_max_/nm	*φ*	*τ*/μs	*k*_r_/10^4^ s^–1^	*k*_nr_/10^4^ s^–1^	*T*_d_/°C	Ref.
[Pt(O^N^N^O)]	**14**	CH_2_Cl_2_	586	0.6	5.3	11.3	7.5	440	70
	**15**	CH_2_Cl_2_	595	0.1	1.9	5.3	47.4	530	70
	**16**	Benzene	546	0.27	3.9	6.9	18.7	382	72
	**17**	Benzene	628	0.26	3.1	8.4	23.9	415	72
	**18**	CH_3_CN	625	0.27	4.62	5.8	15.8	—^*d*^	73
	**19**	CH_2_Cl_2_	661	0.033	1.6	2.1	60.4	—^*d*^	73
	**20**	CH_2_Cl_2_	624	0.20	6.3	3.2	12.7	—^*d*^	74
	**21**	CH_2_Cl_2_	568	0.17	6.34	2.7	13.1	401	75
[Pt(N^N^N^N)]	**22**	CH_3_CN	566, 613 (sh)	0.097	4.2	2.3	21.5	288	76
	**23**	CH_2_Cl_2_	461, 487, 521	0.82	7.8	10.5	2.3	—^*d*^	78
	**24**	CH_2_Cl_2_	520	0.88	2.9	30.3	4.1	—^*d*^	78
[Pt(C^N^N^C)]	**25**	CH_2_Cl_2_	492, 520, 564 (sh)	0.58	0.32	181	131	—^*d*^	79
	**26**	2-MeTHF	512, 548	0.73	7.6^*b*^	9.6	3.6	—^*d*^	80
[Pt(N^C^C^N)]	**27**	2-MeTHF	613	0.14	7.6	1.8	11.3	—^*d*^	80
	**28**	2-MeTHF	486, 516	0.63	5.7	11.0	6.5	—^*d*^	80
	**29**	Bebq_2_ ^*a*^	621	0.58	5.5	10.5	7.6	—^*d*^	81
[Pt(C^C^N^N)]	**30**	CH_2_Cl_2_	660	0.001	0.85	0.12	118	—^*d*^	85
	**31**	CH_2_Cl_2_	550	0.17	4.4	3.9	18.9	—^*d*^	85
[Pt(O^C^C^O)]	**32**	THF	443, 459	0.18	3.5	5.1	23.4	410	87
	**33**	THF	461	0.08	1.8	4.4	51.1	400	87
	**34**	THF	463	0.43	10.0	4.3	5.7	375	—^*e*^
	**35**	THF	465	0.37	11.5	3.2	5.5	—^*d*^	—^*e*^
[Pt(O^N^C^N)]	**36**	CH_2_Cl_2_	485, 517, 557	0.72	12.0	6.0	2.3	414	90
	**37**	CH_2_Cl_2_	488, 518	0.93	13.2	7.0	0.5	406	90
	**38**	CH_2_Cl_2_	515	0.99	15.5	6.4	—^*c*^	—^*d*^	37
	**39**	CH_2_Cl_2_	528, 568	0.74	25.3	2.9	1.0	—^*d*^	37
	**40**	CH_2_Cl_2_	503	0.73	4.7	15.5	5.7	518	91
	**41**	CH_2_Cl_2_	522	0.9	4.9	18.4	2.0	405	91
	**42**	CH_2_Cl_2_	551	0.90	4.3	20.9	2.3	423	93
	**43**	CH_2_Cl_2_	517	0.80	5.1	15.7	3.9	412	93
	**44**	CH_2_Cl_2_	553, 587	0.86	6.6	13.0	2.1	400	92
	**45**	CH_2_Cl_2_	526	0.47	5.9	8.0	9.0	410	92
	**46**	CH_2_Cl_2_	527	0.49	8.8	5.6	5.8	409	92
[Pt(N^C^C^N)]	**47**	CH_2_Cl_2_	454 (sh), 478	0.71	3.3	21.5	8.8	—^*d*^	94
[Pt(N^C^C^C)]	**48**	CH_2_Cl_2_	452	0.78	4.2	18.6	5.2	—^*d*^	94
[Pt(N^C^C^N)]	**49**	CH_2_Cl_2_	430, 456	0.39	3.0	13.0	20.3	—^*d*^	95
[Pt(N^C^C^C)]	**50**	CH_2_Cl_2_	442	0.07	0.4	17.5	232	—^*d*^	95
[Pt(N^C^C^N)]	**51**	CH_2_Cl_2_	513	0.63	2.0	31.5	18.5	—^*d*^	95
	**52**	CH_2_Cl_2_	491	0.81	12.9	6.3	1.5	—^*d*^	97

^*a*^6 wt% doped into a thin film of Bebq_2_.

^*b*^The lifetime *τ*_0_ at infinite dilution determined from the linear variation of the observed emission decay rate constant, *k*_obs_, as a function of the concentration of the complex.

^*c*^Value too low to be reported.

^*d*^Value not available from the literature.

^*e*^The work that has not been reported.

The device doped with **33** at 4 wt% in the host of 9-(4-*tert*-butylphenyl)-3,6-bis(triphenylsilyl)-9*H*-carbazole (CzSi) afforded blue phosphorescence with CIE coordinates of (0.19, 0.21).^89^ The maximum EQE, current efficiency, power efficiency, and brightness were 15%, 23.8 cd A^–1^, 16.6 lm W^–1^ and 9500 cd m^–2^, respectively.^89^ The EL spectrum was stable in the operating voltage of 6–11 V. In combination with a co-deposited yellow phosphorescent Pt(ii) complex, a white light-emitting electro-phosphorescent device was obtained with CIE coordinates, maximum brightness, and current efficiency of (0.32, 0.42), 30 000 cd m^–2^, and 88 cd A^–1^. This work showed that by including an NHC motif in the tetradentate ligand scaffold, efficient deep blue phosphorescent Pt(ii) materials can be generated.

Based on the comparisons between the above-mentioned tetradentate Pt(ii) complexes (Table 2), it is conceived that coordination geometry with short Pt–C bonds and the presence of a fused 6-membered ring may be advantageous in the design of robust, strongly luminescent Pt(ii) emitters. Recently Che and co-workers devised an asymmetric O^N^C^N ligand system that features a metallacycle having fused 6,5,5-membered rings (selected examples **36–39**).^37,90^ The decomposition temperature of this series of complexes is higher than 400 °C (Table 2). All complexes exhibit vibronic-structured emission in CH_2_Cl_2_ and the emitting state has been assigned as metal-perturbed ^3^π–π* of the O^N^C^N ligand. The emission quantum yields of this series of Pt–O^N^C^N complexes are higher than 0.65 and the emission energy can be tuned from 485 to 528 nm. Complexes **36–39**, bearing rigid fluorenyl-like moieties in their O^N^C^N frameworks have small *k*_nr_ values in the range of 0.06–2.3 × 10^4^ s^–1^. This is in contrast to the related Pt(ii) complexes without the dialkylmethylene bridge, which generally have *k*_nr_ values >2.0 × 10^4^ s^–1^ (*vide infra*). Notably, **38** exhibits an emission efficiency of almost one (*φ* = 0.99) in solution at room temperature.^37^ Attachment of bulky *N*-carbazolyl groups to the O^N^C^N ligand scaffold to give **38** results in a significant suppression of intermolecular interactions leading to the fabrication of high-efficiency (EQE = 15.6%) monochromic polymer light-emitting diodes (PLEDs).^37^

Complexes **40** and **41** are related analogues of **36–39**, bearing O^N^C^N ligand scaffolds but without a dialkylmethylene bridge.^91^ The former complexes are also strongly emissive (*φ* = 0.73–0.90 in CH_2_Cl_2_) and thermally stable (*T*_d_ > 400 °C) (Table 2). It is worth noting that the *k*_r_ and *k*_nr_ values for **40** and **41** are higher than those of **36–39**. Consequently, the lifetimes of the former are shortened to fewer than 5 μs while high emission efficiencies are maintained. The bulky norbornene group in **41** was observed to suppress intermolecular interactions, thus disfavouring excimer formation and self-quenching in solution. The high emission efficiency, good thermal stability and ineffective self-quenching property altogether rendered highly phosphorescent OLEDs at a high doping concentration of **41**. The device doped with 13 wt% of **41** in 1,3-bis(*N*-carbazolyl)benzene (mCP) exhibited green phosphorescence with a maximum EQE and peak current efficiency of 18.2% and 66.7 cd A^–1^, respectively. These values are among the highest values for green OLEDs using Pt(ii) emitters. It should be noted that this device showed a very low efficiency roll-off of only 2.4% at 1000 cd m^–2^ (65.1 cd A^–1^).
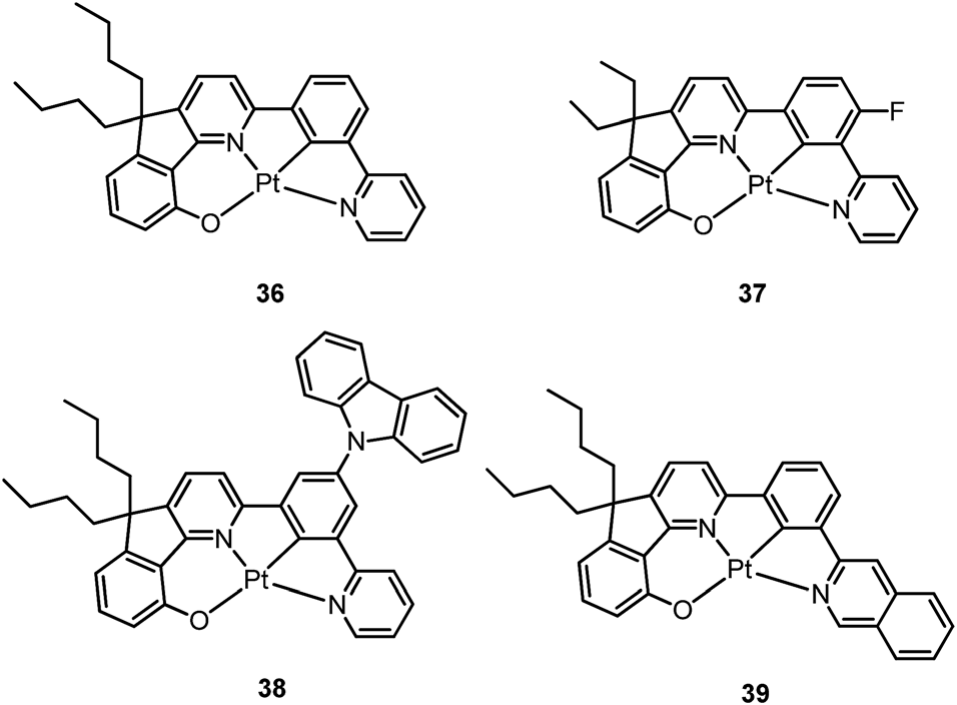


Che has further expanded this O^N^C^N ligand system by inclusion of an additional bridging atom to form fused 6,5,6-membered rings (*e.g.* **42–46**).^92,93^ Complexes **42–46** exhibit strong emission (*φ* = 0.47–0.90 in CH_2_Cl_2_) in the yellow-to-green spectral region (Table 2). Contrary to **44** which has a sterically unencumbered ligand scaffold, **42** and **43** have a bulky orthogonal bridging tertiary amine unit and a biphenyl group with a spiro-linkage respectively; these structural motifs efficiently suppress intermolecular interactions, which is in agreement with both the X-ray crystal structure of **43** and the optimized geometry of the dimer of **42** and **43** using DFT calculations.^93^ As a result, the emission self-quenching rate constants (*k*_q_) are as low as 2.0 × 10^7^ and 1.1 × 10^7^ M^–1^ s^–1^, respectively. With an optimized device structure, maximum power efficiencies of 118 and 126 lm W^–1^ have been achieved with the respective yellow-emitting **42**- and green-emitting **43**-based OLEDs. These values are the highest among the reported Pt(ii)-OLEDs; the maximum EQE were respectively 26.0% and 27.6%.^93^ The emission property of **44** is comparable to that of **42**, revealing that the emitting state is localized on the O^N^C^N motif without significant involvement of the tertiary amine linkage.^92^ The monochromic OLED using **44** as the dopant exhibited a power efficiency of 52.1 lm W^–1^ which was comparable to those of the best Ir(iii)-based yellow OLEDs. In a modified device structure comprising a composite blue host and **44**, a WOLED was obtained with an estimated power efficiency of 61 lm W^–1^. In degassed CH_2_Cl_2_, complexes **45** and **46** show moderate emission quantum yields of 0.47 and 0.49, which are relatively lower than those of **42** and **44**.

Li and co-workers have developed a series of Pt(ii) complexes containing tetradentate ligands of which the conventional cyclometalated fragment C^N is bridged by an O atom to a chelating L^L′ ancillary ligand, resulting in a metallacycle having fused 6,6,5-membered rings (*e.g.* **47–51**).^94,95^ Complexes **47** and **48** having an ancillary pyridyl-carbazole ligand display intense blue phosphorescence in CH_2_Cl_2_ with *λ*_max_ (*φ*) of 478 nm (0.71) and 452 nm (0.78), respectively.^94^ Interestingly, the full-width at half-maximum (FWHM) of **47** decreases from 85 nm to 20 nm upon attaching an electron-donating ^*t*^Bu group to the 4-position of the pyridyl ring.^96^ Similar spectral narrowing has also been observed for **48**. It was conceived that the highly rigid ancillary carbazolyl pyridine motif serves to suppress structural distortion between the triplet emitting state and the ground state, leading to high efficiency emission for this system of Pt(ii) emitters. Moreover, ensuring the localization of the T_1_ state on the designed lumophore ligand by this design strategy serves to suppress vibrational progressions of the triplet emitting state, leading to emission spectra with small Huang–Rhys factors. In addition to a bridging oxygen atom, Li and co-workers have developed a related system in which pyrazolyl-carbazole was selected to function as the lumophore ligand, as shown in **52**.^97^ This new ligand design imposes further rigidity on the complex because of the conjugated nature of the bridging carbazolyl unit within **52**. As a result, a green emission (*λ*_max_ = 491 nm) with improved efficiency (*φ* = 0.81) and a narrow band (FWHM = 18 nm) was achieved for **52**. High-efficiency blue OLEDs with respective EQE of 25.2% and 23.7% were obtained based on **47** and **48**.^94^ Using a ^*t*^Bu-derivative of **48** as the dopant, a highly efficient (EQE = 24.8%) pure blue OLED with CIE coordinates of (0.15, 0.08) was developed.^96^ A green OLED based on **52** demonstrated a high maximum EQE of 25.6% as well as a very small efficiency roll-off (EQE = 25.5% at 100 cd m^–2^).^97^ Complexes **49–51**, having more flexible frameworks, show less intense emission in solution.^95^ In CH_2_Cl_2_, the emission quantum yields of **49** and **51** are 0.39 and 0.63 while that of **50** becomes significantly low (0.07). In PMMA, **51** emits with an efficiency of almost one in the green spectral region. A green phosphorescent OLED using **51** as the dopant showed a maximum EQE of 22.3% which was comparable to that of a *fac*-Ir(ppy)_3_-based device (EQE = 23.6%) with the same device structure.
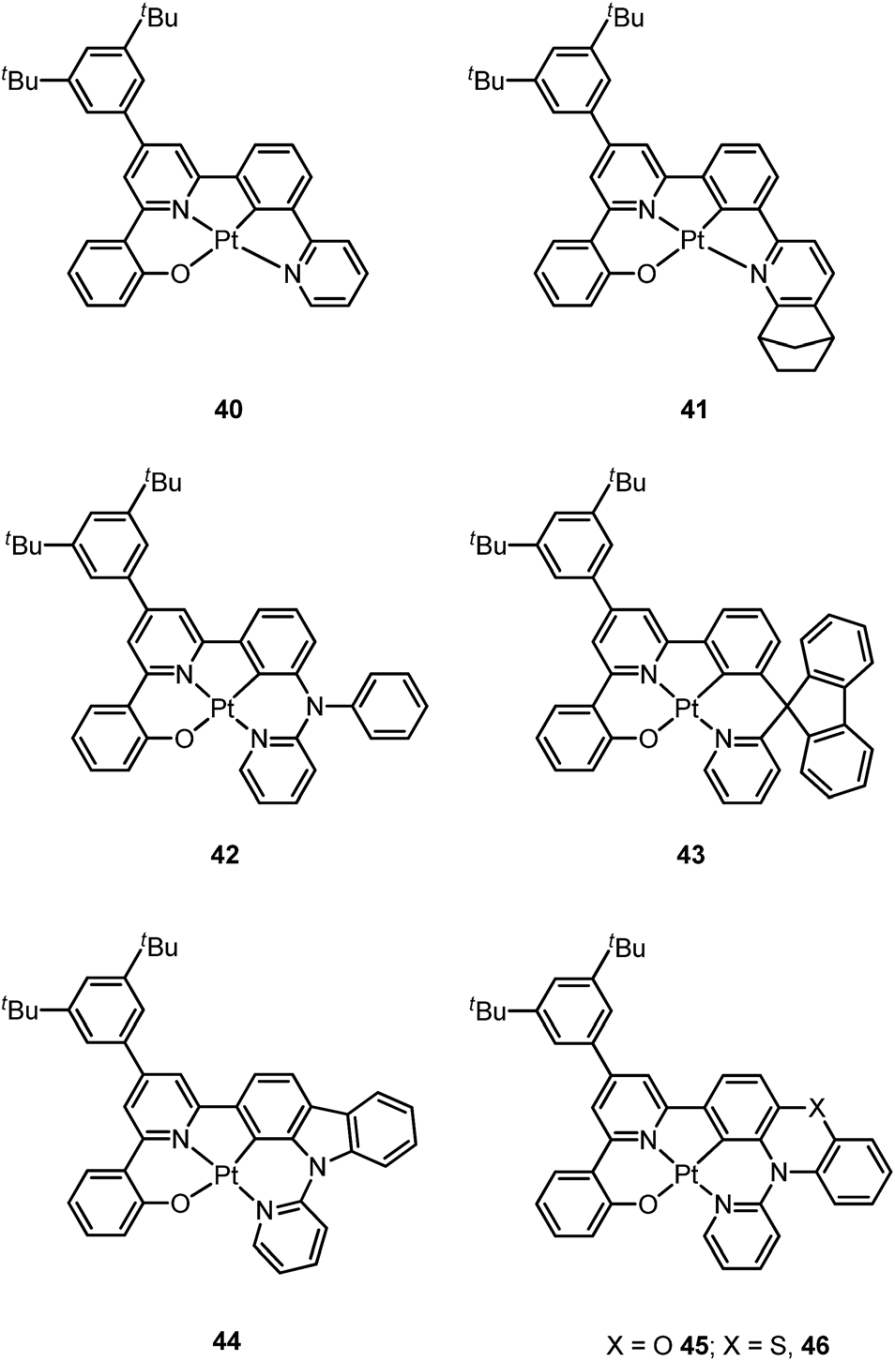
 
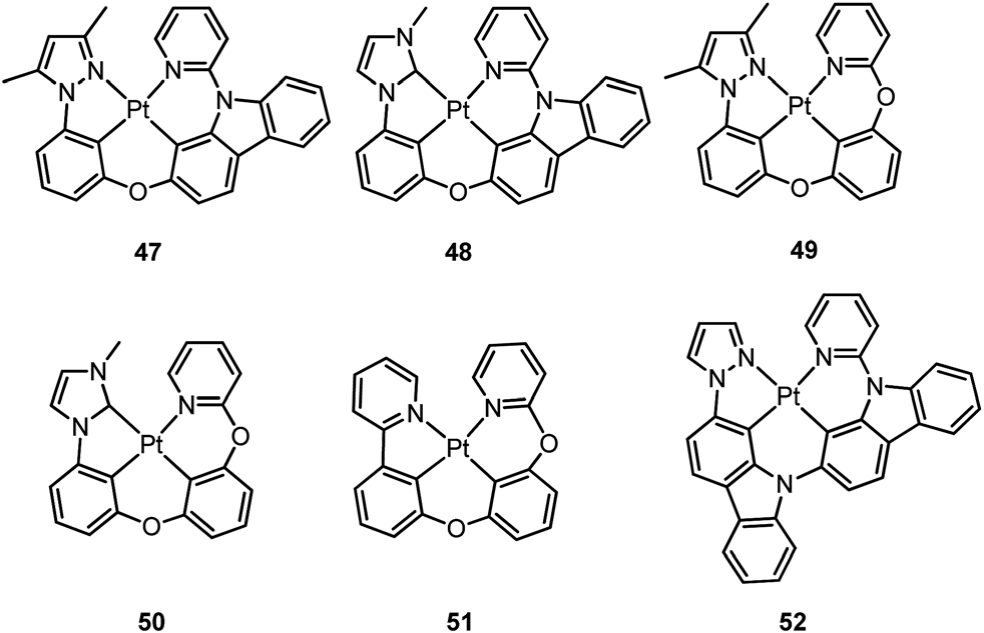


## 4 Self-assembly of luminescent platinum(ii) complexes with material applications

**Scheme 3:**
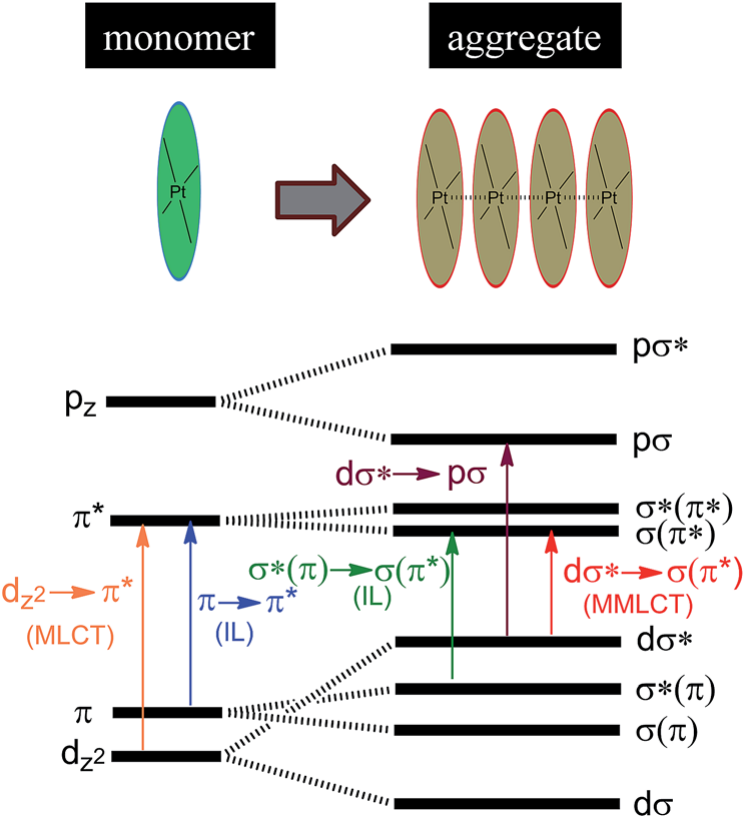
Proposed molecular orbital diagram illustrating d^8^–d^8^ and π–π interactions in Pt(ii) complexes by Miskowski and co-workers in ref. 101.

Luminescent Pt(ii) complexes containing sterically undemanding π-conjugated ligand(s) are prone to associate with each other, driven by intermolecular Pt···Pt and/or π–π interactions (Scheme 3). Miskowski *et al.* pioneered the spectroscopic studies on Pt(ii) complexes of aromatic diimines^8^ and terpyridines^98,99^ revealing that the absorption and emission energies in the solid state are significantly red-shifted in the presence of intermolecular Pt···Pt and/or π–π interactions. The low energy excited states have been termed metal–metal-to-ligand charge transfer (^3^MMLCT) states. Che and co-workers first reported that a dinuclear Pt(trpy) complex with a rigid guanidine bridge displays a moderately intense absorption at 483 nm and weak phosphorescence at 620 nm in degassed CH_3_CN; both phenomena were attributed to the MMLCT excited states.^100^ According to Miskowski and co-workers, the axial 5d_*z* ^2^_ orbitals of two Pt(ii) ions in close proximity overlap to give bonding dσ and antibonding dσ* orbitals (Scheme 3).^101^ Similarly, the π and π* orbitals localized on the π-conjugated ligand can also interact with each other, producing bonding σ(π) and σ(π*) and antibonding σ*(π) and σ*(π*) orbitals. As a consequence, a dσ* → σ(π*) (MMLCT) transition (Scheme 3) occurs with a reduced energy gap, accounting for the red-shifted absorption and emission maxima. Other than MMLCT excited states, σ*(π)–σ(π*) transitions (Scheme 3), as a result of intermolecular π–π interactions, may also lead to electronic excited states of relatively low energy.^102^ Importantly, intermolecular Pt···Pt and/or π–π interactions can occur not only between two ground state molecules but also between an excited state molecule and a ground state one, resulting in lower-energy emissive excimeric ^3^MMLCT or ^3^[σ*(π)–σ(π*)] (usually simplified as ^3^π–π*) excited states.^44^

The intriguing spectroscopic properties dictated by the unique intermolecular Pt···Pt and/or π–π interactions between two ground state molecules or between an excited state molecule and a ground state one can be harnessed for material applications including: (1) excimeric emission for single-dopant WOLEDs; (2) formation of superstructures with optoelectronic applications.

### 4.1 Single-dopant WOLEDs

**Fig. 8:**
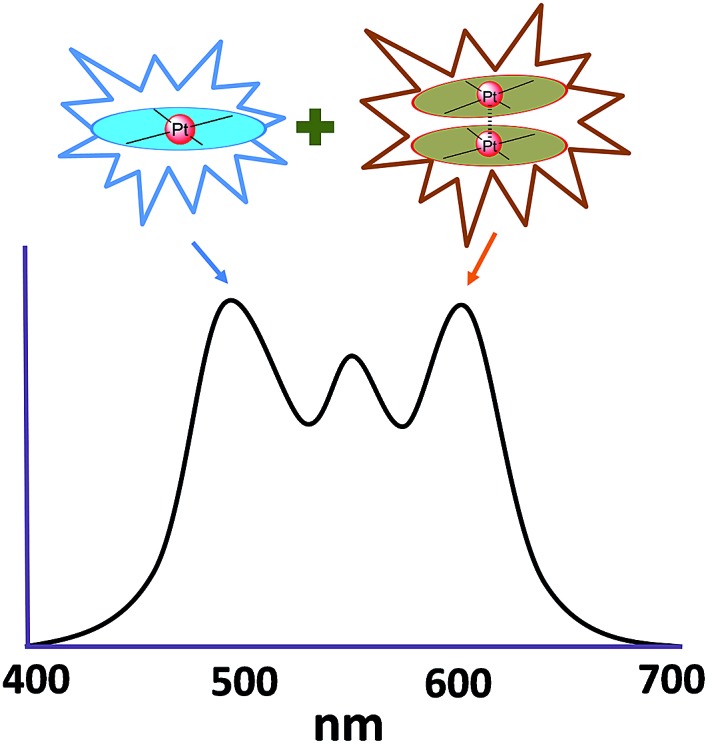
Schematic illustration of the combination of monomeric emission and excimeric emission to produce white light.

In principle, when doped at an appropriate concentration into a host material, the high-energy (in the blue to bluish green region) monomeric and low-energy (in the yellow to red region) excimeric emissions from Pt(ii) emitters can be produced simultaneously, giving rise to a white-light emission (Fig. 8). In contrast to WOLEDs that typically comprise three primary colours (*i.e.* red, green, and blue) or two complementary emission colours (blue and yellow) from multiple phosphorescent emitters, single-dopant WOLEDs offer several advantages.^103^ First, precise control of the relative amount of each dopant for achieving an optimal cascade energy transfer within the emitting layer having multiple dopants is not necessary as only one Pt(ii) dopant is used. Satisfactory white-light emission can be reached by adjusting the concentration of the single Pt(ii) dopant that displays both highly efficient monomeric and excimeric emissions. Second, EL spectral aging due to different dopant aging processes can be excluded as only one molecular light-emitting species is involved, rendering a stable EL spectrum with long-term operation. In the literature, the first excimer-based single-dopant phosphorescent WOLED was demonstrated using **53**.^104^ This complex was reported to display a blue monomeric and orange excimeric emission when doped into a host matrix.^104,105^ Later, pincer-type Pt(ii) complexes **54** and **55**, containing N^C^N ligands, were examined for this purpose.^58,106^ Complexes **54** and **55** show efficient blue phosphorescence (*λ*_max_ = 453–472 nm) with quantum yields of 0.60–0.80 in dilute CH_2_Cl_2_, which is in stark contrast to **53** (*φ* = 0.02 in CH_2_Cl_2_).^42^ However, for all the complexes **53–55**, white EL with a high efficiency and satisfactory CIE as well as CRI were not obtained due to either inefficient monomer emission or inappropriate excimer emission. In 2013, Li and co-workers reported the first high-performance single-dopant WOLED with an EQE > 20%, a satisfactory CIE of (0.33, 0.33), and a CRI of 80 using the pincer-type **56** supported by an NHC-based cyclometalated ligand.^107^ Recently, the EQE of a single-dopant WOLED was boosted to over 25% by Li's group and Che's group by using **57**, **58** or **60** as the emitter.^93,108^ Complex **57** displays efficient blue emission in CH_2_Cl_2_ at *λ*_max_ = 471 nm with a quantum yield and lifetime of 0.77 and 3.2 μs, respectively.^108^ This complex also shows very efficient excimeric emission at elevated concentrations when doped into a host material. As a consequence, the WOLED singly doped with **57** exhibited a peak EQE of 25.7%. A series of related Pt(ii) emitters with sterically un-congested ligands, with **58** as an example, have been prepared; these complexes show both monomeric and excimeric emissions.^109^ Complex **59** exhibits a ^3^π–π*(O^N^C^N) centred bluish-green emission (*λ*_max_ = 482, 512 nm; *φ* = 0.75) in dilute CH_2_Cl_2_.^90^ At elevated concentrations (up to 1 × 10^–4^ M), the emission intensity at 480–520 nm decreases with a concomitant increase in excimeric emission with a maximum at 620 nm. An efficient WOLED was fabricated using **59** as a single dopant. The peak EQE, current efficiency, and power efficiency were 16.5%, 71.0 cd A^–1^, and 55.8 lm W^–1^, respectively.^90^ Since steric bulkiness of the alkyl chains on the fluorene moiety may affect the intermolecular interactions, the butyl chains in **59** have been replaced with ethyl chains to facilitate intermolecular interactions in **60**.^37^ Very recently, the EQE and power efficiency of the vacuum-deposited WOLED singly doped with **60** were reported to be 25.1% and 55.5 lm W^–1^ respectively in an optimized device structure.^93^ Che and co-workers also examined the EL performances of **59** and **60** in solution-processed white polymer OLEDs. A peak EQE, current efficiency, and power efficiency of 9.7%, 17.0 cd A^–1^, and 9.1 lm W^–1^, respectively, were observed for **59**.^90^ For **60**, the EQE was 12.73%, which slightly decreased to 11.51% at a high brightness of 1000 cd m^–2^, revealing a very low efficiency roll-off.^37^ The high EQE obtained for vacuum-deposited or solution-processed WOLEDs based on **57–60** suggests that Pt(ii) emitters with highly efficient monomeric and excimeric emissions at appropriate energies are highly promising candidate materials for WOLEDs. However, for all WOLEDs singly doped with **57–60**, the CIE (*y* > 0.41) and CRI < 80 revealed an unsatisfactory quality of the white light output. These could be attributed to an insufficiently high energy of the blue monomer emission. Hence, the development of highly efficient deep blue emitting Pt(ii) complexes with structures that favour highly emissive excimer formation is crucial for attaining high-quality single-dopant WOLEDs.
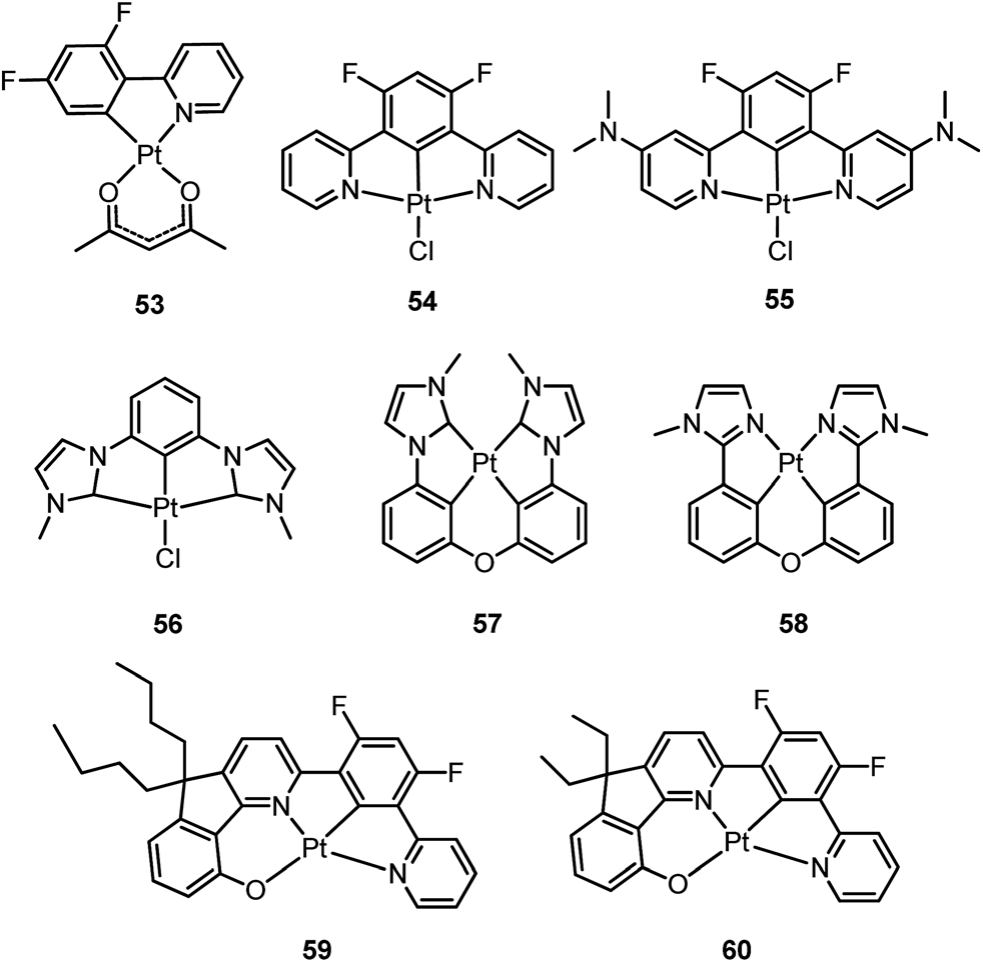


### 4.2 Self-assembled functional molecular materials

**Scheme 4:**
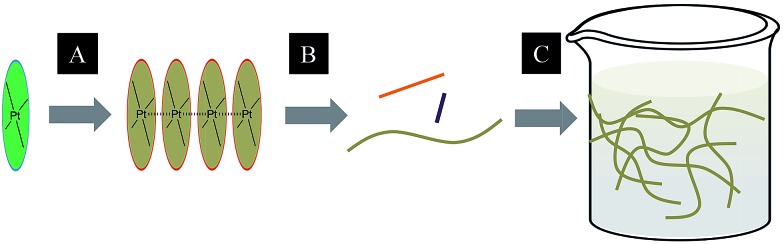
The illustration of self-assembly of Pt(ii) complexes into 1D nanostructures and soft materials.

**Fig. 9:**
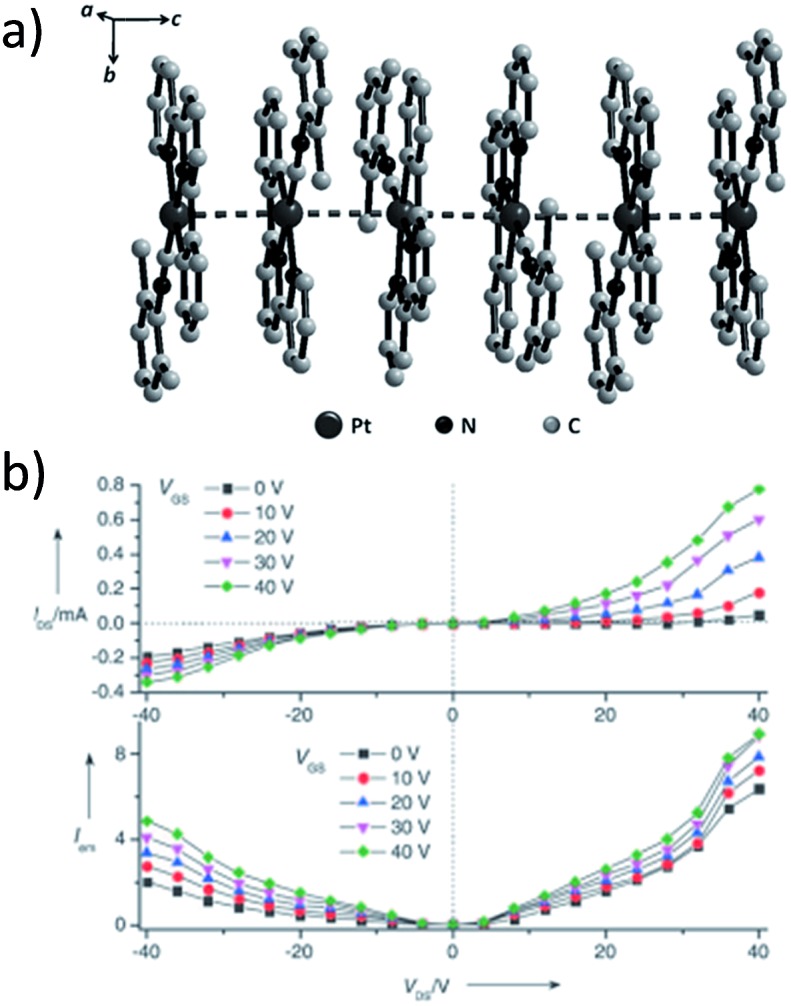
(a) Crystal packing diagram of **61**·H_2_O. The PF_6_^–^ and water molecules are omitted for clarity. (b) Output characteristics (*I*_DS_ *vs. V*_DS_) and electroluminescence intensity (*I*) of an OLEFET device with nanowires of **61** after annealing at 350 K (adapted with permission from ref. 112. Copyright 2008, Wiley-VCH Verlag GmbH & Co. KGaA).

**Fig. 10:**
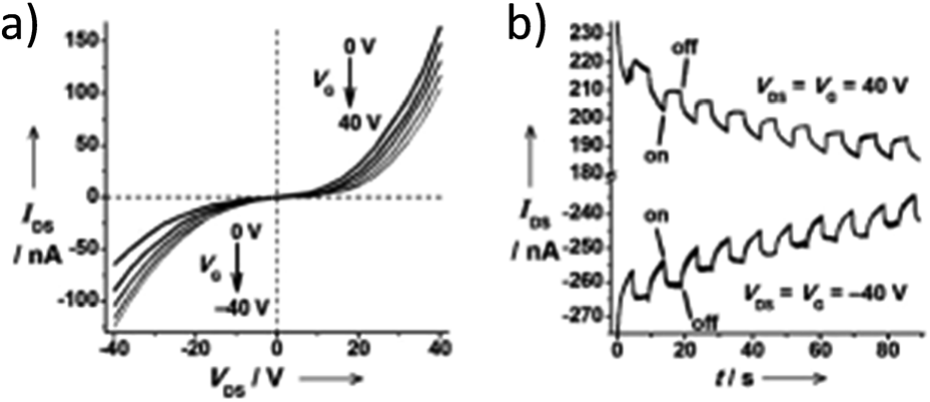
(a) Output characteristics (*I*_DS_ *vs. V*_DS_) of a FET device with nanosheets of **66**. (b) Transient measurement (*V*_DS_ = *V*_G_ = ± 40 V) of the FET device. The transient channel current was recorded with a 40 mW cm^–2^ light switching on and off every five seconds in a 90 second period. *I*_DS_ = drain-source current, *V*_DS_ = drain-source voltage, *V*_G_ = gate voltage (adapted with permission from ref. 123. Copyright 2009, Wiley-VCH Verlag GmbH & Co. KGaA).

A subject of growing interest is the self-assembly of phosphorescent Pt(ii) complexes into functional molecular materials. This bottom-up approach is illustrated in Scheme 4: (A) self-assembly of Pt(ii) complexes into polynuclear aggregates, and (B) further aggregation into quasi-1D nano- or micro-structures (nanowire, nanorod, nanofiber, *etc.*),^110–116^ which can, in certain cases, (C) organize into soft materials such as gels.^115,117–122^ In comparison to the self-assembly of pure organic systems, the presence of phosphorescent Pt(ii) units can provide a spectroscopic handle to follow the self-assembling process based on the high sensitivity of the emission of Pt(ii) complexes to Pt···Pt and π–π interactions. Furthermore, the presence of Pt···Pt and Pt(ii)–organic ligand interactions can enhance the stability of the resultant soft material. Due to the luminescent properties and highly ordered stacking arrangements, the resulting Pt(ii)-superstructures with optoelectronic properties can be used for potential applications in materials science. In this context, we and others have explored the self-assembly behavior of a series of luminescent pincer-type Pt(ii) complexes. For instance, we showed that cationic Pt(ii) complex **61** can pack in a highly ordered manner with alternate Pt···Pt distances of 3.382 and 3.344 Å. The nanowires self-assembled from **61** display semiconducting properties and phosphorescence, permitting the fabrication of organic light-emitting field-effect transistors (OLEFETs) (Fig. 9).^112^ Che and Lu recently reported the synthesis of a strongly emissive Pt(ii) allenylidene complex **63**, which was found to form nanorods *via* self-assembly in CH_3_CN/H_2_O.^113^ Interestingly, transformation from nanorods to nanorings upon increasing the water fraction in the solvent mixture was observed.^113^ Charge transport is conceived to be facilitated along highly ordered one-dimensional Pt···Pt and π–π packing chains. A high field-effect electron mobility of up to 20 cm^2^ V^–1^ s^–1^ was recorded with a transistor fabricated from a single microcrystal of **64**.^114^ De Cola and co-workers developed a neutral Pt(ii) emitter, **65**, supported by dianionic tridentate terpyridine-like ligand. This complex is non-emissive in dilute solution and undergoes self-assembly to give gelating nanowires accompanied by a striking phosphorescence switch-on (*φ* = 0.90).^115^ Apart from the 1D nano- and micro-structures, Che and co-workers showed that the neutral Pt(ii) complex **66** underwent self-assembly into quasi-2D nanosheets driven by the orthogonal Pt···Pt and C–H···π(C≡C) interactions. These nano-sheets showed NIR phosphorescence and visible light-modulated electronic conductivity (Fig. 10).^123^ 
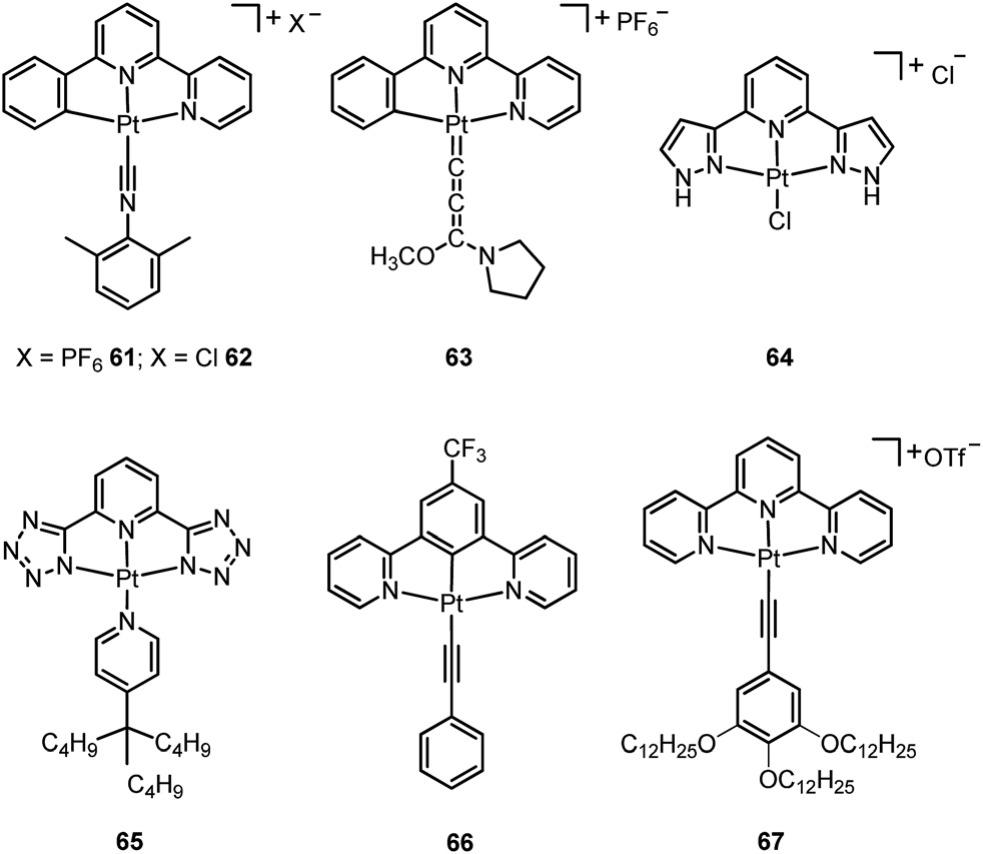


**Fig. 11:**
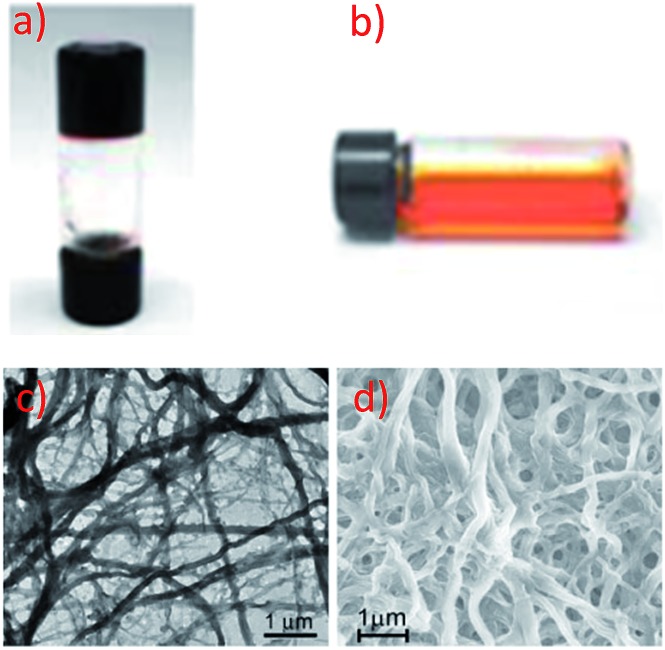
Photographs of **67** in DMSO in (a) the gel form at room temperature, (b) the sol form at elevated temperature. (c) TEM and (d) SEM images of the xerogel of **67** formed in DMSO (adapted from ref. 118 with permission from the Royal Society of Chemistry).

Yam and co-workers have extensively studied supramolecular soft materials based on luminescent alkynylplatinum(ii) complexes with tridentate N-donor ligands, particularly terpyridine.^124^ It was reported that **67** can form a metallo-gel in DMSO with a low critical gelation concentration of 4.4 mg mL^–1^.^118^ Drastic colour and emission changes were observed upon the sol-to-gel transition (Fig. 11a and b), revealing that Pt···Pt and π–π interactions were involved in the gel formation. Transmission electron microscopy (TEM) and scanning electron microscopy (SEM) images of the xerogel showed a network of fibrous structures with a diameter and length of approximately 470 nm and 10 μm, respectively (Fig. 11c and d). Unlike the supramolecular soft materials formed from organic molecules bearing H-bonding motifs and/or long soft alkyl chains,^125^ the Pt···Pt and π–π interactions between organoplatinum(ii) units provide the driving force for self-assembly. On the other hand, the anion of cationic Pt(ii) complexes has been found to play a crucial role in determining both the extent of intermolecular interactions and the interactions with the solvent medium, thereby providing an entry for tuning the softness of the self-assembled structures.^126,127^ As a result, simple and easy-to-synthesize Pt(ii) molecules, not containing H-bonding motifs and long alkyl chains, can be used as building blocks for supramolecular soft materials. For example, **62**, an analogue of **61** with a Cl^–^ anion, is soluble in water and was observed to form a chromic meso-phase in water with a critical concentration of 1.5 wt%.^119^

**Fig. 12:**
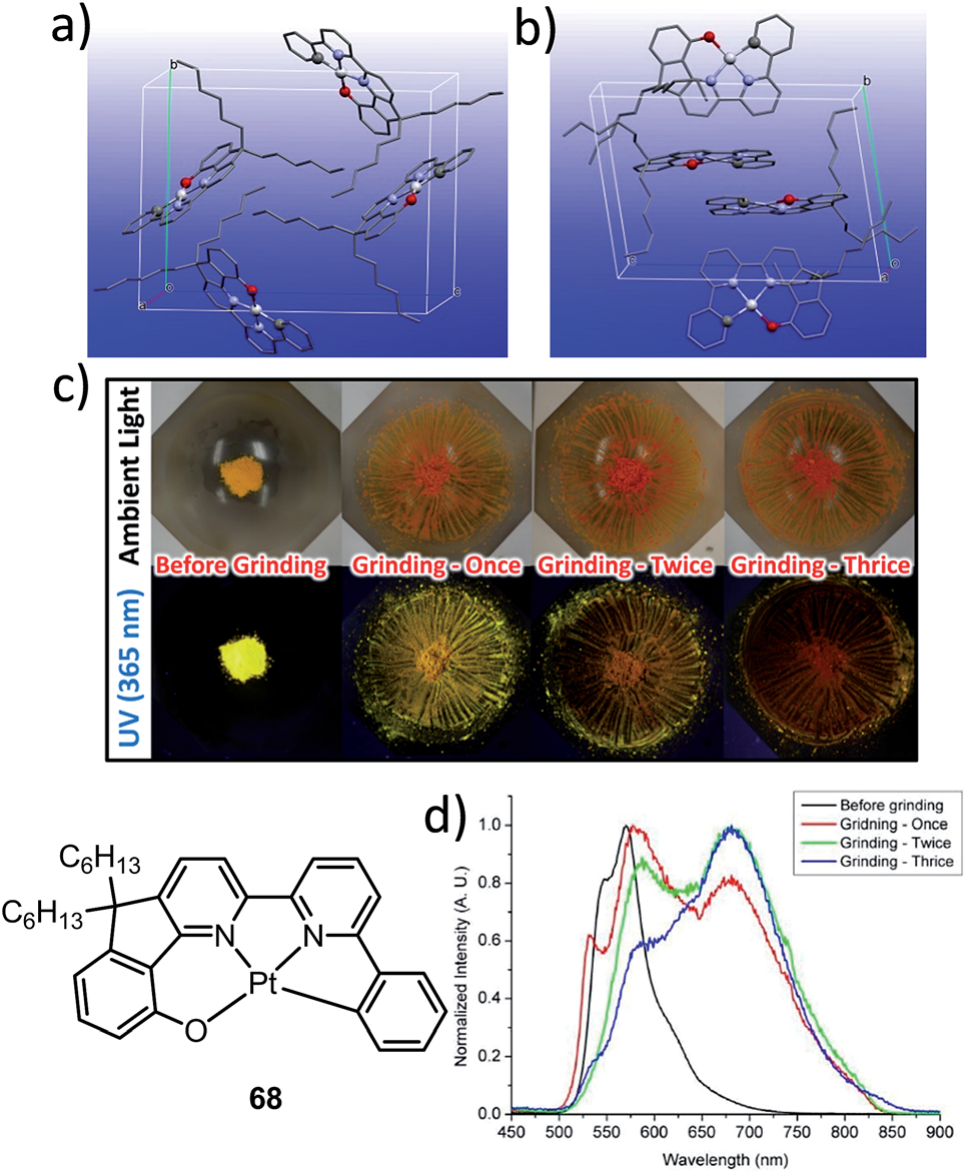
Crystal packing of **68** in (a) monoclinic form and (b) triclinic form. (c) Photographs of the yellow crystalline solid of **68** before and after grinding under ambient and UV light irradiation (365 nm). (d) Emission spectra of **68** (yellow triclinic form) before and after grinding.

The manipulation of the molecular packing arrangement in the solid state by external mechanical stimuli renders Pt(ii) emitters^127–131^ as promising mechanoluminescent (ML) candidates for the development of sensory and data storage materials. We synthesized another new type of luminescent Pt(ii) complex, **68**, with tetradentate O^N^N^C ligands (Scheme S2, ESI†). Complex **68** is an analogue of the Pt(ii)–O^N^C^N complex **36**. Yellow and orange crystal polymorphs of **68** in monoclinic and triclinic forms, respectively, were obtained by slow evaporation of a CH_2_Cl_2_ solution (Fig. 12a and b). The intermolecular distances between adjacent molecules in the monoclinic and triclinic crystal forms are 3.524 and 3.468 Å, respectively, revealing the presence of weak π–π interactions. There are no Pt···Pt interactions in either case, since the determined closest Pt···Pt distance is longer than 4.8 Å. The yellow crystalline solid (triclinic form) precipitated from CH_2_Cl_2_/hexane displays a vibronic-structured emission (*λ*_max_ = 541, 571, 621 (sh) nm). Upon grinding, the emission peak first shifted to 588 nm and then a broad emission band at 681 nm was obtained (Fig. 12c and d). This red-shift of emission is probably due to enhanced intermolecular Pt···Pt and/or π–π interactions. Addition of Et_2_O or another solvent to the ground solid restored the colour and emission spectrum of the yellow crystalline solid. Interestingly, soaking the ground sample with Et_2_O for about 2 h resulted in an orange crystalline solid, of which the PXRD pattern was in line with the simulated pattern of the monoclinic crystal form. The interconversions between different forms of **68** were confirmed by PXRD studies (Fig. S5, ESI†). To the best of our knowledge, **68** represents the first example of a Pt(ii) complex supported by a tetradentate ligand that shows polymorphic and mechanochromic luminescence properties.

**Fig. 13:**
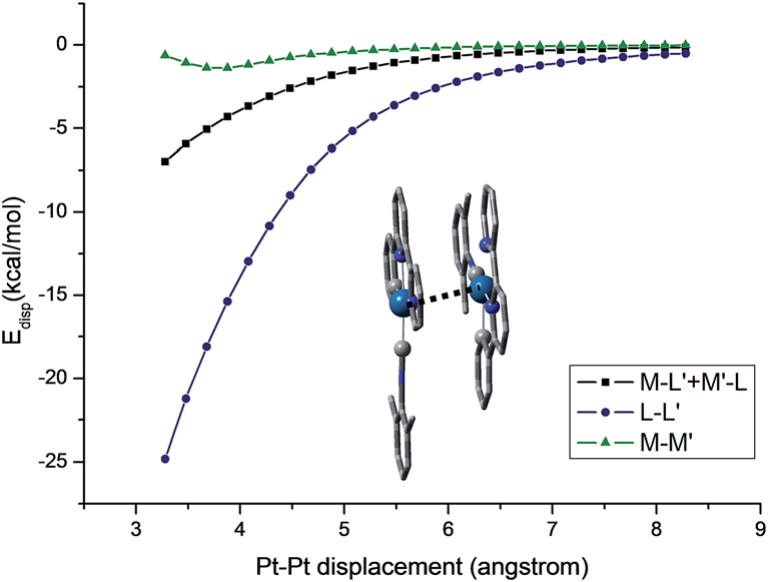
Dispersion contributions of the metal atoms and ligand fragments to the dimer [**61**–PF_6_]_2_^2+^. The Pt···Pt′ distance was used as a reaction coordinate.

The self-organization of d^8^ transition metal complexes, such as those of Rh(i), into luminescent functional assemblies constitutes a fascinating topic within supramolecular chemistry because of their unique photophysical properties imparted by metal···metal interactions.^132,133^ The main driving forces have been generally attributed to the interplay of intermolecular metal···metal and/or π–π interactions (Scheme 3).^134^ In this context, the nature of the intermolecular interactions and the contribution of each interaction component, such as metallophilicity, to the stabilization of aggregates has been an ongoing debate due to the difficulty in accessing relatively high-level theory for computational studies.^135–139^ Recently, a dispersion-corrected DFT method has been shown to be a viable approach to provide a quantitative assessment of the contribution of each interaction component to the intermolecular stabilizing energy of d^8^ Rh(i) and d^10^ Au(i) dimers.^139,140^ It was shown that the d^8^···d^8^ interactions of Rh(i) dimers only account for a small fraction (10–15%) of the dispersion contribution to the total binding energy when π-conjugated ligands are present.^139^ In the case of relatively large Au(i) systems, the weak ligand–ligand interaction dominates dimer formation.^140^ The role of dispersion interactions in driving the self-assembly of luminescent Pt(ii) systems has not been studied and its interplay with other weak non-covalent interactions in determining the morphology of the self-assembly structures has not been defined. This prompted us to examine the nature and weight of Pt···Pt interactions by studying the dimer of pincer-type Pt(ii) complex **61** which has a strong tendency to aggregate (Fig. 9).^112^ As depicted in Fig. 13, at a Pt···Pt distance of 3.28 Å, the dispersive Pt···Pt, Pt···ligand and ligand···ligand interactions amount to 0.62, 6.98, and 24.81 kcal mol^–1^, respectively, revealing that the London dispersive attraction dominates the dimer formation of the Pt(ii) molecules.

Inclusion of spin–orbit coupling (SOC) is also essential to understanding the photophysical properties of d^8^–d^8^ diplatinum complexes. Recently, spin–orbit TDDFT (SO-TDDFT) calculations were performed on [Pt_2_(*μ*-P_2_O_5_H_2_)_4_]^4–^ (abbreviated as Pt(pop)) and its perfluoroborated derivative, [Pt_2_(*μ*-P_2_O_5_(BF_2_)_2_)_4_]^4–^ (abbreviated as Pt(pop-BF_2_)) to shed light on the ISC mechanism in these complexes.^141^ It was highlighted that the ^1^dσ*pσ → ^3^dσ*pσ ISC is facilitated by second-order spin–orbit coupling with the high-lying ligand-to-metal–metal charge transfer (LMMCT) dπpσ and pπpσ excited states. Owing to the calculation results that Pt(pop-BF_2_) has a larger energy gap between the dσ*pσ and the LMMCT excited states, Pt(pop-BF_2_) has a slower ISC rate than Pt(pop). It was also proposed that the structural flexibility of Pt(pop) facilitates transient distortions which allows enhanced spin–orbit coupling (SOC) between the ^1^dσ*pσ and ^3^dσ*pσ excited states, leading to a much faster ISC rate in Pt(pop). As a result, Pt(pop) shows dominant phosphorescent emissions but dual fluorescence-phosphorescence is observed for Pt(pop-BF_2_).

## 5 Conclusion and outlook

Multidentate ligands containing strong σ-donor atoms are advantageous for the construction of robust, phosphorescent Pt(ii) emitters. The strong σ-donor strength and high rigidity of the ligand scaffold can push the d–d states well above the triplet emitting state thereby minimizing excited state structural distortion and non-radiative decay. Due to strong chelate effect, the stability of Pt(ii) complexes can be significantly improved with the use of multidentate ligands. Following these design principles, a number of phosphorescent Pt(ii) complexes that are photo-stable, kinetically inert and chemically stable under various conditions have been developed. Using terdentate or tetradentate cyclometalating ligands, tremendous progress has been made in tuning the emission energy and emission quantum yield as well as excited state lifetime of Pt(ii) complexes. OLEDs based on luminescent Pt(ii) complexes emanating red, yellow, green, or blue light with maximum EQEs higher than 20% have been reported. The unique excimeric emission from luminescent Pt(ii) complexes has been harnessed in the fabrication of efficient single-dopant WOLEDs. Luminescent Pt(ii) complexes have also been shown to have rich photochemistry; for example, they act as efficient photocatalysts for organic transformations. Intermolecular Pt···Pt and π–π interactions provide directional driving forces for the anisotropic growth of 1D or 2D nano- or microstructures with interesting optoelectronic properties.

The planar coordination geometry also endows the phosphorescent Pt(ii) complexes with axial coordination sites for metal–substrate interactions, forming an operating principle for luminescent sensory applications such as cellular imaging. Numerous Pt(ii) terpyridyl (trpy) complexes have been recognized to be effective DNA and RNA intercalators, owing to the presence of a planar Pt-trpy moiety that favours π–π stacking interactions with the base pairs of nucleic acids.^142,143^ However, Pt(ii) terpyridyl complexes usually suffer from inferior luminescence properties. To circumvent the d–d state-induced emission quenching of Pt(ii) terpyridyl complexes, physiologically stable luminescent Pt(ii) complexes supported by a cyclometalated ligand or tetradentate ligand were developed and studied.^144–146^ The binding of these complexes with bio-molecules were observed to induce elevations of emission intensity and/or shifts of emission maxima, as a result of the reduced excited state geometry distortion and/or the formation of new emissive adducts. In this context, the good kinetic stability of Pt(ii) complexes with tetradentate ligands in solution, together with their luminescent properties, makes them highly promising bio-sensors.

**Fig. 14:**
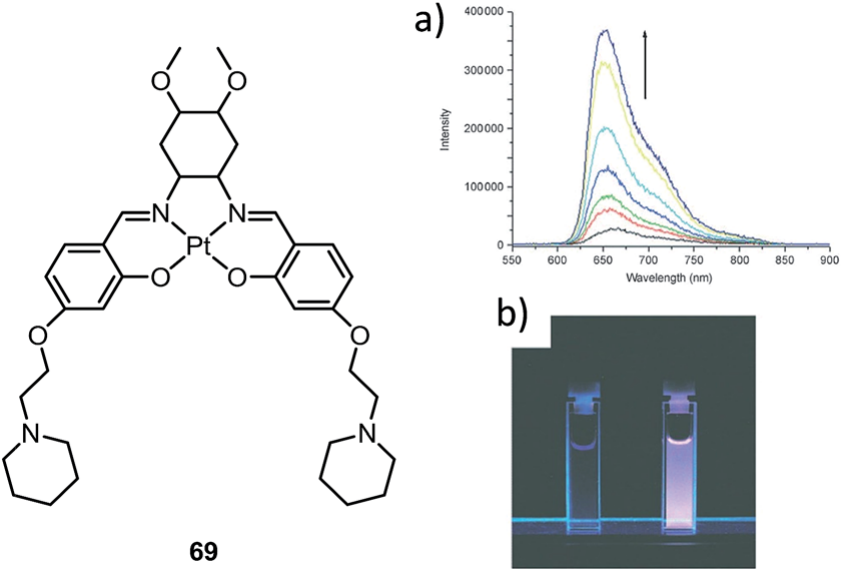
(a) Emission spectral traces of **69** (50 μM) in Tris/KCl buffer with increasing concentrations of G-quadruplex DNA at 20 °C. (b) Photographs of **69** in the absence or the presence of G-quadruplex DNA (adapted with permission from ref. 147. Copyright 2009, Wiley-VCH Verlag GmbH & Co. KGaA).

Che and co-workers described their molecular design studies of a Pt(ii) Schiff base complex **69** containing peripheral amine side chains as a c-*myc* G-quadruplex DNA binder.^147^ Based on UV-Vis absorption and NMR spectroscopic measurements, this complex is stable in aqueous solution for 72 h at room temperature. Complex **69** is weakly emissive in aqueous Tris/KCl buffer solution. Upon addition of the G4A1 quadruplex DNA, an intense emission at 652 nm developed and the emission intensity increased 8-fold at a [G-quadruplex]/[**69**] ratio ≥ 20 (Fig. 14). By absorption titration experiments, the binding constant was determined to be 1.72 ± 0.26 M^–1^ at 20 °C, which is approximately ten-fold of that with non-quadruplex double-stranded DNA molecules. An external end-stacking mode between **69** and G-quadruplex DNA was suggested based on the findings from NMR titration experiments and molecular modeling studies. To the best of our knowledge, this represents the first system of a Pt(ii) complex supported by tetradentate ligands as a luminescent bio-probe.

**Fig. 15:**
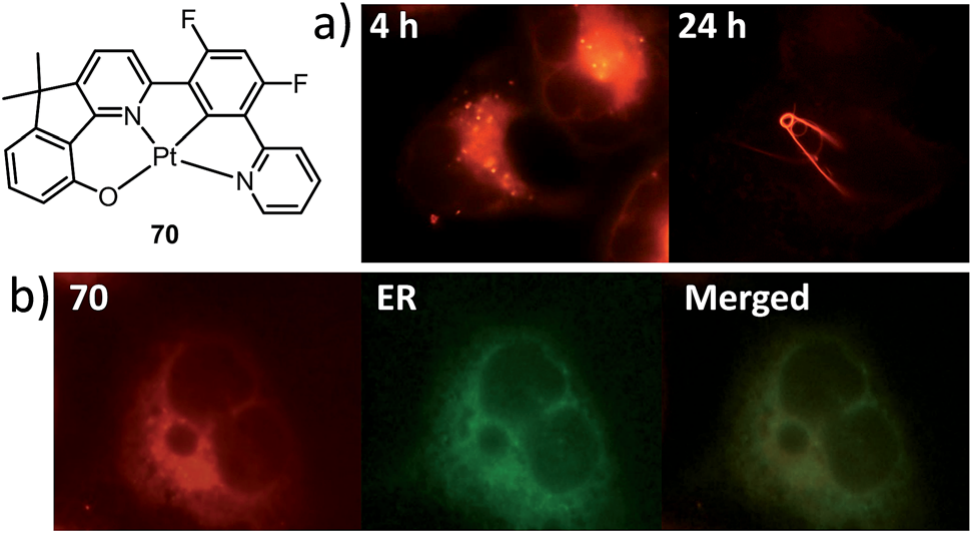
Fluorescence imaging of cells treated with **70**. (a) Imaging of HeLa cells treated with **70** (5 μM) for 4 and 24 h (*λ*_ex_ = 440 nm; *λ*_em_ > 590 nm). (b) Co-localization of **70** and ER-RFP (red fluorescent protein) revealed that the complex selectively accumulates in the ER. Cells were treated with **70** (5 μM) for 2 h.

Complex **70** has been reported to display an intense bluish-green emission at low concentrations (<10^–5^ M in CH_2_Cl_2_) (*λ*_max_ = 479, 510 nm) while giving an intense excimeric red emission (*λ*_max_ = 624 nm) at elevated concentrations close to 10^–4^ M.^37^ Complex **70** was found to aggregate into micron sized rod-like structures in both organic and aqueous media (Fig. S6, ESI†). These rods showed a strong red emission under ambient conditions. We examined the cell-imaging properties of **70**. Cervical epithelioid carcinoma (HeLa) cells treated with **70** initially displayed a strong orange emission, revealing the presence of both monomeric and excimeric emissions from **70**. Interestingly, the formation of an emissive rod-aggregate was observed in the cellular environment after a 12 h treatment with **70**. No obvious morphological change was identified after a 24 h treatment at 10 μM, suggesting **70** is of low cytotoxicity. The co-staining analysis revealed that the complex selectively accumulates in the endoplasmic reticulum (ER) prior to aggregate formation (Fig. 15). Hence, our results show that **70** localizes in ER and subsequently self-assembles into a rod-like morphology. The self-assembled superstructures of luminescent Pt(ii) complexes have been recently recognized to afford advantages in bioimaging applications because of their stable and persistent emission by shielding oxygen- and solvent molecule-induced emission quenching, and their more biocompatible low-energy emission.^148–150^ Herein, we have demonstrated the application of a highly luminescent Pt(ii) complex with a tetradentate ligand in cell-imaging and, more importantly, the control of the self-assembly behaviour of exogenous molecules in a cellular environment.

Despite the striking advances that have been made in the development of robust, efficient Pt(ii) emitters, there are fundamental issues that need to be resolved prior to their practical applications. These issues include: (1) the limited number of Pt(ii) complexes that display efficient deep blue emission. In view of their importance in full-colour display and white-light illumination and in the design of new strongly oxidizing and reducing excited state species for visible light-driven organic transformations, a deep understanding of the structure–photophysics relationship of, and further development of deep blue emitting Pt(ii) complexes are required; (2) the pursuit of stable NIR emitting Pt(ii) complexes for medical applications because of the biocompatibility and high transmittance of NIR photons; (3) the small number of Pt(ii) complexes reported to display efficient excimeric emissions. As the structural features affecting the photophysical properties of excimeric emissions are not well understood, the rational design and exploitation of excimeric emissions of luminescent Pt(ii) complexes remains a formidable challenge. Nevertheless, robust and highly phosphorescent Pt(ii) complexes would have tremendous applications in diverse disciplines. In addition to OLED applications, the practical utilization of luminescent Pt(ii) complexes, supported by tetradentate ligands, as robust cell imaging agents, new photocatalysts, and building blocks for self-assembled functional molecular materials has a bright future.

## Note added after first publication

This article replaces the version published on 7th January 2016, which contained errors in the caption for Fig. 5.
