# Supplementary material for: Highly phosphorescent platinum(ii) emitters: photophysics, materials and biological applications
Source: Chem Sci. 2016 Jan 7;7(3):1653–73. doi: 10.1039/c5sc03766b (PMC6090519; doi:10.1039/c5sc03766b)
Supplement: Supplementary file 1 [file SC-007-C5SC03766B-s001.pdf]

Electronic Supplementary Material (ESI) for Chemical Science.
This journal is © The Royal Society of Chemistry 2015

Electronic Supplementary Information

Highly phosphorescent platinum(II) emitters: photophysics, materials and biological applications

Kai Li,[ab] Glenna So Ming Tong,[a] Qingyun Wan,[a] Gang Cheng,[ab] Wai-Yip Tong,[a] Wai-Hung Ang,[a] Wai-Lun Kwong[a] and Chi-Ming Che[*ab]

[a] *State Key Laboratory of Synthetic Chemistry, Institute of Molecular Functional Materials, HKU-CAS Joint Laboratory on New Materials, and Department of Chemistry, The University of Hong Kong, Pokfulam Road, Hong Kong, China. E-mail: cmche@hku.hk*

[b] *HKU Shenzhen Institute of Research and Innovation, Shenzhen 518053, China*

Experimental Section

Materials, reagents and instrumentation

All materials used were received from commercial sources unless stated otherwise. Elemental analyses were performed by the Institute of Chemistry at the Chinese Academy of Sciences, Beijing. Fast atom bombardment (FAB) mass spectra were obtained on a Finnigan Mat 95 mass spectrometer. The ^{1}H, ^{13}C, and ^{19}F spectra were recorded on DPX 300, Avance 400 or DRX 500 Bruker FT-NMR spectrometers. The solvents used for photophysical measurements were of HPLC grade. UV-vis spectra were recorded on an Agilent / HP 8453 diode array spectrophotometer. Steady-state emission spectra at 298 K were obtained on a Spex 1681 Flurolog-2 Model F111 spectrophotometer equipped with a Hamamatsu R928 PMT detector. All solutions for photophysical measurements, except stated otherwise, were degassed in a high-vacuum line with at least four freeze-pump-thaw cycles. Emission lifetimes were measured with a Quanta-Ray Q-switch DCR-3 Nd:YAG pulsed laser system. Emission quantum yields of solutions were measured using a degassed acetonitrile solution of a solution of quinine hemisulfate salt monohydrate in 0.5 M H_2SO_4 (ϕ_r = 0.546) as the standard and calculated by $\phi_s = \phi_r(B_r/B_s)(n_s/n_r)^2(D_s/D_r)$, where the subscripts s and r refer to the sample and reference standard solution respectively, n is the refractive index of the solvents, D is the integrated intensity, and ϕ is the luminescence quantum yield. The quantity B is calculated by $B = 1–10^{-AL}$, where A is the absorbance at the excitation wavelength and L is the optical path length. Errors for wavelength values (1 nm) and ϕ (10%) are estimated.

Single crystals of **68** (monoclinic) and **68** (triclinic) suitable for X-ray diffraction analysis were obtained from CH_2Cl_2/MeOH or by slow evaporation of a CH_2Cl_2 solution, respectively. The diffraction data were collected on a Bruker X8 PROTEUM single crystal X-ray diffractometer with MicroStar rotating-anode X-ray source (CuKa radiation, λ = 1.5418 Å). CCDC-1008480 and CCDC-1008481 contain the supplementary crystallographic data for **68** (monoclinic) and **68** (triclinic), respectively. These data can be obtained free of charge from The Cambridge Crystallographic Data Centre via www.ccdc.cam.ac.uk/data_request/cif.

Transmission electron microscopy (TEM) analysis

Suspensions of **70** in CH_2Cl_2/hexane (10^{-4} M, v/v; 1:1) and DMSO/H_2O (10^{-5} M, v/v; 1:9) were transferred to carbon-coated copper grids and completely dried under vacuum. The samples were analyzed by FEI Tecnai G2 20 S-Twin Scanning Transmission Electron Microscope. The TEM images were taken by Gatan MultiScan Camera Model 794.

Cellular imaging

HeLa cells were maintained in Eagle's minimum essential medium supplemented with 10% v/v fetal bovine serum, L-glutamine (2 mM), and penicillin/streptomycin (100 U/mL). HeLa cells (2 $\times10^5$) were seeded on 35-mm glass bottomed microwell dishes and treated with **70** (5 μM). The cells were observed under a Zeiss Axiovert 200M inverted fluorescence microscope (λ_{ex} = 440 nm; λ_{em} > 590 nm). For the co-localization study, the cells were transfected using the plasmid DNA of YFP-ER (0.4 μg/mL, Yrbio) for 24 h prior to the treatment of the platinum complex.

Synthesis and characterization of 34–35 and 68

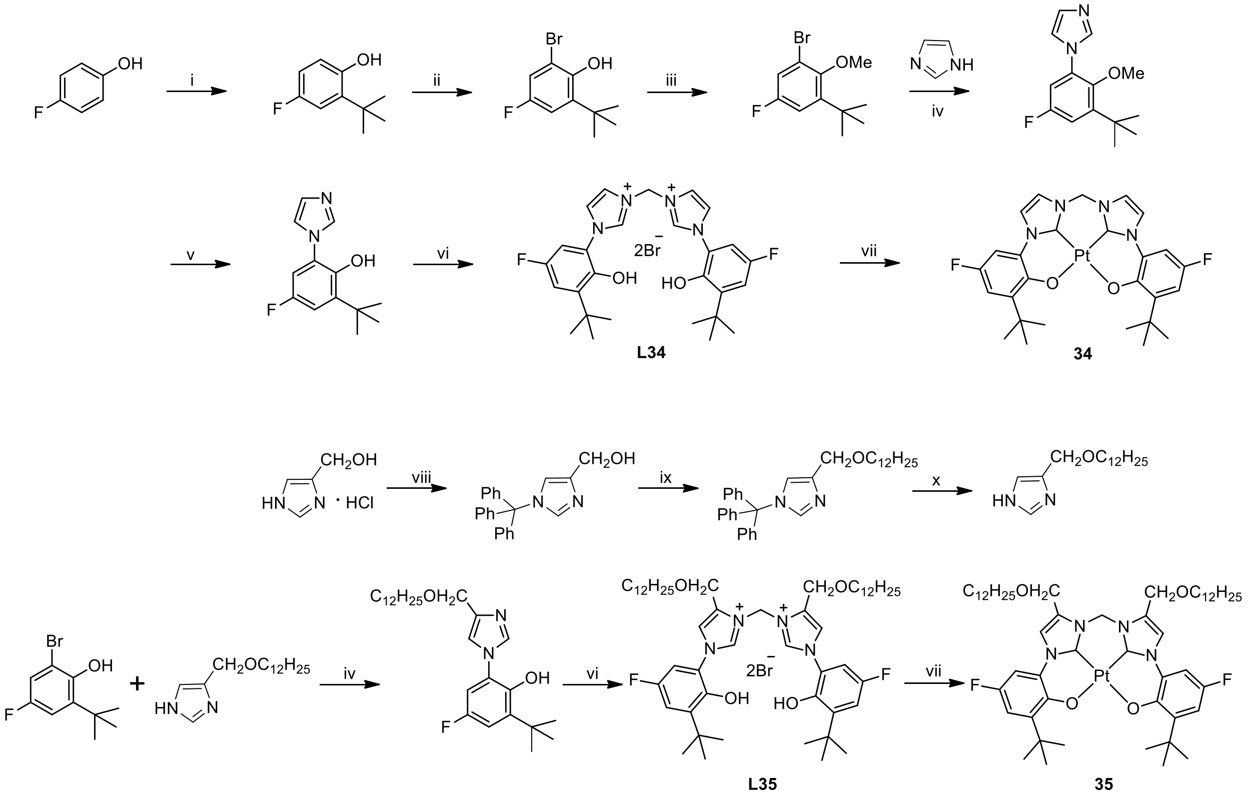

Scheme S1 Synthetic routes to complexes **34** and **35**. Reagents and conditions: (i) Conc. H_2SO_4, ^{t}BuOH, 0 °C to r.t.; (ii) NBS, $^i Pr_2NH$, reflux; (iii) MeI, K_2CO_3, r.t.; (iv) CuI, L-proline, K_2CO_3, 120 °C; (v) Pyridinium chloride, 170 °C; (vi) CH_2Br_2, 110–140 °C; (vii) $Pt(DMSO)_2Cl_2$, Et_3N, 80 °C; (viii) Et_3N, Ph_3CCl; (ix) NaH, $^n C_{12}H_{25}Br$, KI; (x) TFA, r.t..

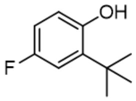

2-(tert-Butyl)-4-fluorophenol

To a chilled solution of 4-fluorophenol (5.66 g, 50 mmol) and *tert*-butyl alcohol (4.81 mL, 50 mmol) in CH_2Cl_2 (50 mL) was added concentrated H_2SO_4 (2.81 mL). The solution was allowed to warm to ambient temperature and stirred for 48 h. Then, another aliquot of *tert*-butyl alcohol (4.81 mL, 50 mmol) and concentrated H_2SO_4 (2.81 mL) were added. The solution was further stirred at ambient temperature for 48 h. The solution was then treated with saturated $NaHCO_3$ (aq.) until evolution of gas ceased. The mixture was then extracted with CH_2Cl_2. The organic layer was washed with $NaHCO_3$ (aq.) and brine and dried over Na_2SO_4. After filtered and concentrated, the crude product was purified via flash column chromatography on silica-gel with eluent of hexane, affording light yellow liquid with almost quantity yield. ^{1}H NMR (400 MHz, $CDCl_3$) δ (ppm) 6.97 (dd, J = 10.9 Hz, 2.9 Hz, 1H), 6.77-6.72 (m, 1H), 6.60 (dd, J = 8.6 Hz, 4.9 Hz, 1H), 4.87 (s, 1H), 1.39 (s, 9H); ^{19}F NMR (376 MHz, $CDCl_3$) δ (ppm) –124.01.

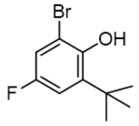

2-Bromo-6-(tert-butyl)-4-fluorophenol

To a refluxing solution of 2-(*tert*-butyl)-4-fluorophenol (7.3 g, 43.3 mmol) and diisopropylamine (0.61 mL, 4.35 mmol) in 150 mL CH_2Cl_2 was added NBS (7.7 g, 43.3 mmol) portionwise through Soxhlet apparatus under nitrogen. The solution was refluxed for 24 h during which time the NBS was slowly consumed. After cooling to room temperature, the mixture was treated with 2 M sulfuric acid (130 mL). The crude product was purified via column chromatography on silica-gel with eluent of hexane, affording the desired product as a pale yellow liquid (6.74 g, 63%). ^{1}H NMR (300 MHz, $CDCl_3$) δ(ppm) 7.08 (dd, J = 7.0, 2.9 Hz, 1H), 6.98 (dd, J = 10.6, 2.8 Hz, 1H), 5.60 (s, 1H), 1.38 (s, 9H); ^{19}F NMR (376 MHz, $CDCl_3$) δ (ppm) –122.29.

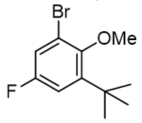

2-Bromo-6-(tert-butyl)-4-fluoroanisole

A mixture of 2-bromo-6-(*tert*-butyl)-4-fluorophenol (3.20 g, 12.94 mmol) and iodomethane (0.95 mL, 15.26 mmol) in DMF (25 mL) with the presence of K_2CO_3 (2.68 g, 19.37 mmol) was sealed and stirred at room temperature in dark for 24 h. After completion of the reaction, the mixture was poured into 100 mL water and then extracted with hexane (50 mL × 3). The combined organic fractions were dried over Na_2SO_4. After removing solvent, the residue was filtered through a short plug of silica-gel with eluent of hexane, affording light yellow liquid (3.04 g, 90%). ^{1}H NMR (400 MHz, $CDCl_3$) δ (ppm) 7.15 (dd, J = 7.0, 3.1 Hz, 1H), 7.00 (dd, J = 10.6, 2.9 Hz, 1H), 3.90 (s, 3H), 1.38 (s, 9H); ^{19}F NMR (376 MHz, $CDCl_3$) δ (ppm) –117.60.

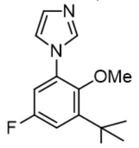

1-(3-(tert-Butyl)-5-fluoro-2-methoxyphenyl)-1H-imidazole

To a degassed DMSO (15 mL) solution of 2-bromo-6-(*tert*-butyl)-4-fluoroanisole (1.56 g, 5.97 mmol) and imidazole (810 mg, 11.90 mmol) were added CuI (120 mg, 0.63 mmol), L-proline (145 mg, 1.26 mmol) and K_2CO_3 (1.78 g, 12.8 mmol) under nitrogen atmosphere. The resulting mixture was stirred at 130 °C for 60 h under N_2. After cooling to room temperature, the mixture was poured into ethyl acetate (50 mL) and was filtered. The filtrate was washed with water (50 mL × 3) and dried over Na_2SO_4. After rotary evaporation, the crude product was purified via column chromatography on silica gel with eluent of ethyl acetate/hexane (1/1, v/v), affording colorless crystalline solid (490 mg, 33%). ^{1}H NMR (300 MHz, $CDCl_3$) δ (ppm) 7.76 (s, 1H), 7.23 (s, 1H), 7.21 (s, 1H), 7.08 (dd, J = 10.5, 3.1 Hz, 1H), 6.89 (dd, J = 7.6, 3.1 Hz, 1H), 3.21 (s, 3H), 1.40 (s, 9H); ^{19}F NMR (376 MHz, $CDCl_3$) δ (ppm) –117.16.

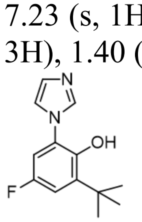

2-(tert-Butyl)-4-fluoro-6-(1H-imidazol-1-yl)phenol

A mixture of 1-(3-(*tert*-butyl)-5-fluoro-2-methoxyphenyl)-1H-imidazole (460 mg, 1.86 mmol) and pyridinium chloride (4.26 g, 36.84 mmol) was refluxed for 4 h under N_2. After cooling to

room temperature, the mixture was treated with water and $NaHCO_3$ (aq.). The precipitate was collected by filtration and purified via flash chromatography with eluent of ethyl acetate, affording white solid (310 mg, 71%). ^{1}H NMR (400 MHz, $CDCl_3$) δ (ppm) 9.03 (s, 1H), 7.29 (s, 1H), 7.14 (dd, J = 10.6, 2.9 Hz, 1H), 7.07 (s, 1H), 6.95 (s, 1H), 6.73 (dd, J = 7.5, 2.9 Hz, 1H), 1.47 (s, 9H); ^{19}F-NMR (376 MHz, $CDCl_3$) δ (ppm) –122.89.

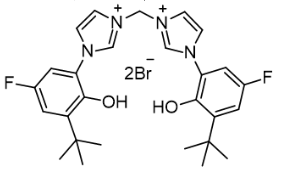

L34

A solution of 2-(*tert*-butyl)-4-fluoro-6-(1H-imidazol-1-yl)phenol (306 mg, 1.31 mmol) and dibromomethane (1.0 mL, 14 mmol) in 5 ml THF was refluxed at 115 °C for 48 h. After cooling to room temperature, the resulting white precipitates was collected by filtration, washed with THF and dried in vacuo (321 mg, 76%). ^{1}H NMR (400 MHz, DMSO-d_6) δ (ppm) 9.91 (s, 2H), 9.47 (s, 2H), 8.23 (s, 2H), 8.13 (s, 2H), 7.38 (d, J = 7.4 Hz, 2H), 7.29 (d, J = 10.6 Hz, 2H), 6.92 (s, 2H), 1.39 (s, 18H). ^{19}F NMR (376 MHz, DMSO-d_6) δ (ppm) –121.37.

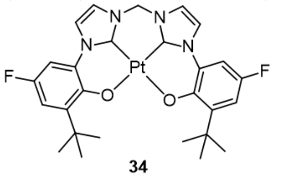

34

A mixture of the ligand precursor **L34** (52 mg, 0.081 mmol), $Pt(DMSO)_2Cl_2$ (34 mg, 0.081 mmol) and Et_3N (0.068 mL, 0.49 mmol) in EtOH (6 mL) was heated to 80 °C for 6 h. After completion of reaction, the mixture was cooled to room temperature, collected by filtration, washed with ethanol, ether and then dried in vacuo, affording an off-white solid (39 mg, 72%). ^{1}H NMR (400 MHz, DMSO-d_6) δ (ppm) 8.32 (s, 2H), 7.67 (s, 2H), 7.56 (d, J = 10.5 Hz, 2H), 6.88 (d, J = 10.6 Hz, 2H), 6.30 (s, 2H), 1.46 (s, 18H); ^{19}F NMR (376 MHz, DMSO-d$_6$) δ (ppm) –130.33; FAB-MS (+ve, m/z) 673 $[M]^+$; Anal. Calcd (%) for $C_{27}H_{28}F_2N_4O_2Pt$: C 48.14, H 4.19, N 8.32; Found: C, 48.04, H 4.29, N 8.21.

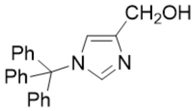

(1-Trityl-1H-imidazol-4-yl)-methanol[1]

To a solution of 4-(hydroxymethyl)-imidazole hydrochloride salt (2.23 g, 16.6 mmol) in DMF (50 mL) was added triethylamine (5.8 mL, 41.6 mmol). After stirred for 10 min, a solution of chlorotriphenylmethane (5.16 g, 18.5 mmol) in DMF (40 mL) was added dropwise. After stirred overnight under a nitrogen atmosphere, the reaction mixture was poured into ice water and the mixture was filtered. The solid was washed with dioxane and dried under vacuum to afford (1-trityl-1H-imidazol-4-yl)-methanol as a white powder (100% yield).

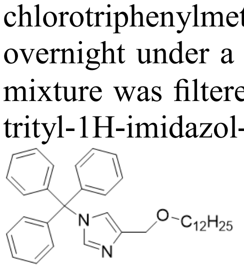

4-((Dodecyloxy)methyl)-1-trityl-1H-imidazole

To a suspension of (1-trityl-1H-imidazol-4-yl)-methanol (5.10 g, 15.0 mmol) in DMF (100 mL) was added NaH (60% in oil) (1.20 g, 30.0 mmol) and the mixture was stirred under N_2 at room

temperature for 0.5 h, followed by addition of 1-docecyl bromide (5.40 mL, 22.5 mmol). The mixture was further stirred at room temperature for 12 h. After reaction, the mixture was poured into H_2O and extracted with ethyl acetate. The combined organic phase was dried over $MgSO_4$ and rotary evaporated after filtration. Further purification by column chromatographic on silica gel with hexane/EA (1:1, v/v) afford white solid (5.51 g, 73%). ^{1}H NMR (400 MHz, $CDCl_3$) δ (ppm) 7.39 (s, 1H), 7.32 (m, 9H), 7.14 (m, 6H), 6.80 (s, 1H), 4.42 (s, 2H), 3.48 (t, J = 6.8 Hz, 2H), 1.58 (m, 2H), 1.24 (m, 18 H), 0.87 (t, J = 6.8 Hz, 3H).

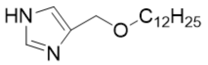

4-((Dodecyloxy)methyl)-1H-imidazole

A solution of 4-((dodecyloxy)methyl)-1-trityl-1H-imidazole (5.21 g, 10.2 mmol) and 35 mL trifluoroacetic acid (TFA) in dichloromethane (150 mL) was stirred at room temperature for 4 h. After reaction, the solution was washed with H_2O (3 × 100 mL), saturated $NaHCO_3$ (aq. 100 mL), and then brine (100 mL). The organic phase was dried over $NaSO_4$ and rotary evaporated to dryness after filtration. Further purification through column chromatography on silica-gel with dichloromethane/MeOH (1:0 to 10:1, v/v) afford white crystalline solid product (2.58 g, 95%). ^{1}H NMR (400 MHz, $CDCl_3$) δ (ppm) 7.60 (s, 1H), 7.00 (s, 1H), 4.49 (s, 2H), 3.47 (t, J = 6.7 Hz, 2H), 1.58 (m, 2H), 1.24 (m, 18 H), 0.87 (t, J = 6.6 Hz, 3H).

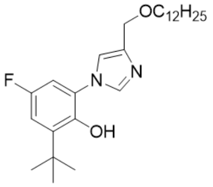

2-(*tert*-Butyl)-6-(4-((dodecyloxy)methyl)-1H-imidazol-1-yl)-4-fluorophenol

A mixture of 4-((dodecyloxy)methyl)-1H-imidazole (2.56 g, 9.61 mmol), 2-bromo-6-(*tert*-butyl)-4-fluorophenol (2.6 g, 10.5 mmol), CuI (190 mg, 1.0 mmol), L-proline (230 mg, 2.0 mmol) and K_2CO_3 (1.63 g, 11.8 mmol) in DMSO (50 mL) was degassed and then stirred at 120 ^oC for 24 h under N_2. After cooling to room temperature, the mixture was poured into 40 mL HCl aq. (2 M) and then basified with saturated $NaHCO_3$ (aq.) to PH = 7.5. The mixture was then extracted with ethyl acetate (20 mL × 3). The combined organic fractions were dried over Na_2SO_4 and then filtered. The filtrate was rotary evaporated and the residue was purified through column chromatography on silica-gel with ethyl acetate/hexane (51:2, v/v) as eluent, affording light yellow oil (0.77 g, 17%). ^{1}H-NMR (400 MHz, $CDCl_3$) δ (ppm) 9.58 (s, 1H), 7.30 (s, 1H), 7.10 (dd, J = 10.6, 3.1 Hz), 7.00 (s, 1H), 6.67 (dd, J = 7.5, 3.1 Hz, 1H), 4.21 (s, 2H), 3.25 (t, J = 6.6 Hz, 2H), 1.57 (m, 2H), 1.27 (m, 27H), 0.88 (t, J = 6.3 Hz, 3H); ^{19}F-NMR (376 MHz, $CDCl_3$) δ (ppm) −123.49.

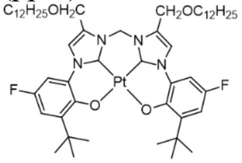

35

A mixture of 2-(*tert*-butyl)-6-(4-((dodecyloxy)methyl)-1H-imidazol-1-yl)-4-fluorophenol (0.39 g, 0.90 mmol) and dibromomethane (1 mL, 14 mmol) in THF (5 mL) was heated at 130 ^oC for 48 h. After reaction, the mixture was rotary evaporated and washed with diethyl ether, affording an oil mixture (0.38 g). The crude product **L35** was used in next step without further purification.

A mixture of the as-obtained crude ligand **L35**, $Pt(DMSO)_2Cl_2$ (0.15 g, 0.36 mmol) and Et_3N (0.3 mL, 2.15 mmol) in EtOH (15 mL) was heated to 80 °C for 6 h. After completion of reaction, the mixture was cooled to room temperature, collected by filtration, washed with ethanol and then dried under vacuum, affording pure product as off-white solid (76 mg; two-step yield: 16%). ^{1}H-NMR (400 MHz, THF-d_8) δ(ppm) 7.93 (s, 2H), 7.20 (d, *J* = 10.4 Hz, 2H), 6.93 (d, *J* = 9.6 Hz, 2H), 6.27 (s, 2H), 4.40 (s, 4H), 3.42 (t, *J* = 5.8 Hz, 4H), 1.73–1.25 (m, 58H), 0.87 (t, *J* = 6.4 Hz, 6H); FAB-MS (+ve, m/z) 1069.4 $[M]^+$. Elemental analysis calcd (%) for $C_{53}H_{80}F_2N_4O_4Pt$: C 59.48, H 7.53, N 5.23. Found: C 60.11, H 7.63, N 5.27.

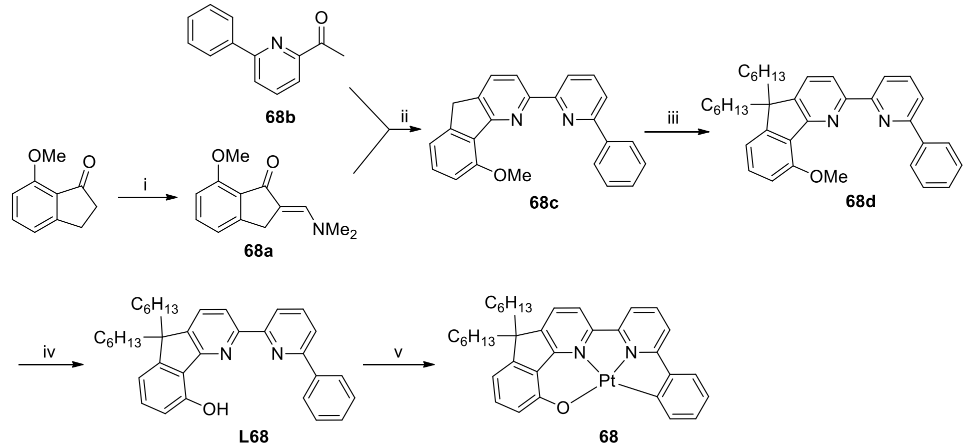

Scheme S2 Synthetic routes to complex **68**.

2-((Dimethylamino)methylene)-7-methoxy-2,3-dihydro-1H-inden-1-one (68a)

7-Methoxyinden-1-one (5.00 g, 30.83 mmol) was dissolved in *N*,*N*-dimethylformamide dimethyl acetal (10 mL). The mixture was refluxed overnight. The volatile organics were then removed under vacuum and the solid formed was washed with cold hexane. The product was obtained as a yellow solid. Yield: 4.91 g (73 %). ^{1}H NMR (400 MHz, $CDCl_3$): δ 7.38–7.42 (m, 2H), 7.27 (s, 1H), 7.00 (d, *J* = 8.1 Hz, 1H), 6.9 (d, *J* = 8.2 Hz, 1H), 3.95 (s, 3H,), 3.84 (s, 2H), 3.15 (s, 6H).

2-Acetyl-6-phenylpyridine (68b)

To a clean 100 mL round bottom flask, 40 mL toluene and 10 mL 2M aqueous potassium carbonate solution was added to a stirred mixture of 1-(6-bromopyridin-2-yl)ethanone (2.02 g, 10.00 mmol), phenylboronic acid (1.34 g, 10.10 mmol) and tetrakis(triphenylphosphine)palladium(0) (0.30 g, 0.26 mmol) under nitrogen atmosphere. The mixture was then refluxed overnight. The crude product was extracted with dichloromethane, and was purified by column chromatography using hexane/dichloromethane (1:1) as eluent. The product was obtained as a white solid. Yield: 1.91 g (97 %). ^{1}H NMR (400 MHz, $CDCl_3$): δ 8.12–8.10 (m, 2H), 7.99–7.87 (m, 3H), 7.54–7.44 (m, 3H), 2.83 (s, 3H).

68c

A mixture of 2-acetyl-6-phenylpyridine (1.91 g, 9.68 mmol) and potassium *tert*-butoxide (1.20 g, 10.69 mmol) in 50 mL THF was stirred overnight under nitrogen atmosphere for 2 h before the addition of 2-((dimethylamino)methylene)-7-methoxy-2,3-dihydro-1H-inden-1-one (2.10 g, 9.67 mmol) dissolved in 30 mL THF through cannula. The reaction mixture was stirred overnight. 30 g ammonium acetate and 70 mL glacial acetic acid were subsequently added and THF was removed by distillation for 2 h. The crude product was taken up by water and extracted using dichloromethane, and purified by column chromatography using hexane/dichloromethane (1:1)

as eluent. The product was obtained as a yellowish white solid. Yield: 1.58 g (46 %). ^{1}H NMR (300 MHz, $CDCl_3$): δ 8.66–8.58 (m, 2H), 8.20–8.17 (m, 2H), 7.93 (t, *J* = 7.9 Hz, 2H), 7.78 (d, *J* = 7.8 Hz, 1H), 7.55–7.38 (m, 4H), 7.22 (d, *J* = 7.5 Hz, 1H), 7.00 (d, *J* = 8.2 Hz, 1H), 4.16 (s, 3H), 3.96 (s, 2H).

68d

Potassium *tert*-butoxide (1.22 g, 10.83 mmol) was added to a stirred solution of **68c** (1.58 g, 4.51 mmol) in 50 mL THF in nitrogen protected atmosphere. The mixture was stirred for 30 min followed by the addition of 1-bromohexane (1.86 g, 11.28 mmol). The reaction mixture was refluxed for 2 h. The crude product was partitioned between water and dichloromethane, and purified by column chromatography using hexane/dichloromethane (2:1) as eluent. Compound **68d** was obtained as a yellowish white solid. Yield: 2.09 g (89%). ^{1}H NMR (300 MHz, $CDCl_3$): δ 8.62 (t, *J* = 7.4 Hz, 2H), 8.18 (d, *J* = 7.2 Hz, 2H), 7.93 (t, *J* = 7.8 Hz, 1H), 7.78–7.74 (m, 2H), 7.54–7.38 (m, 4H), 7.02 (d, *J* = 7.5 Hz, 1H), 6.94 (d, *J* = 8.2 Hz, 1H), 4.15 (s, 3H), 2.09–1.92 (m, 4H), 1.14–0.89 (m, 12H), 0.74 (t, *J* = 6.9 Hz, 6H), 0.66–0.59 (m, 4H).

L68

Ligand precursor **68d** (2.09 g, 4.03 mmol) and 20 g pyridine hydrochloride were added to a clean dry 100 mL round bottom flask. The mixture was degassed by three pump-fill cycles and protected by nitrogen, and then it was heated to melting for more than 2 h. The reaction progress was monitored by TLC. After the consumption of starting material, the reaction was quenched by water when hot. The crude product was extracted by dichloromethane and was purified by column chromatography using hexane/dichloromethane as eluent. The ligand **L68** was obtained as a pale yellow solid. Yield: 0.56 g (27%). ^{1}H NMR (500 MHz, $CDCl_3$): δ 9.38 (s, 1H), 8.56 (d, *J* = 7.9 Hz, 1H), 8.35 (d, *J* = 7.7 Hz, 1H), 8.18 (d, *J* = 7.2 Hz, 2H), 7.93 (t, *J* = 7.8 Hz, 1H), 7.78–7.92 (m, 2H), 7.53 (t, *J* = 7.5 Hz, 2H), 7.46 (t, *J* = 6.3 Hz, 1H), 7.34 (t, J = 7.8 Hz, 1H), 6.91–6.95 (m, 2H), 1.94–2.06 (m, 4H), 1.02–1.27 (m, 12H), 0.69–0.79 (m, 10H). ^{13}C NMR (126 MHz, $CDCl_3$): δ 160.76, 156.58, 155.29, 154.34, 154.01, 152.48, 143.96, 139.38, 137.76, 131.18, 130.95, 129.07, 128.76, 126.99, 124.28, 120.22, 118.98, 118.67, 114.35, 113.49, 54.42, 39.75, 31.48, 29.64, 24.00, 22.53, 13.98. EI-MS (+ve, m/z): 505 [M^+].

Complex **68**

A mixture of ligand **L68** (0.56 g, 1.11 mmol) and K_2PtCl_4 (0.55 g, 1.33 mmol) in glacial acetic acid (90 mL) and chloroform (10 mL) was refluxed for 2 days. The mixture was then cooled to room temperature and partitioned between water and dichloromethane. The crude product was purified by column chromatography using hexane/dichloromethane as eluent. Complex **68** was obtained in 43% yield.

^{1}H NMR (500 MHz, $CDCl_3$): δ 8.01 (d, *J* = 7.6 Hz, 1H), 7.83–7.79 (m, 2H), 7.69–7.63 (m, 2H), 7.55–7.49 (m, 3H), 7.42 (t, *J* = 7.2 Hz, 1H), 7.37 (d, *J* = 8.5 Hz, 1H), 7.16 (t, *J* = 7.5 Hz, 1H), 6.75 (d, *J* = 7.0 Hz, 1H), 2.12–2.01 (m, 4H), 1.15–1.00 (m, 12H), 0.82–0.68 (m, 10H). ^{13}C NMR (126 MHz, $CDCl_3$): δ 168.54, 162.01, 154.62, 153.44, 152.34, 150.46, 150.31, 148.62, 148.26, 137.27, 134.41, 133.03, 131.03, 128.71, 124.99, 124.52, 124.09, 119.65, 117.60, 117.19, 116.28, 109.58, 56.41, 39.81, 31.47, 29.64, 24.20, 22.51, 13.96. FAB-MS (+ve, m/z): 698 [M^+]. Anal. Calcd for $C_{35}H_{38}N_2OPt$: C 60.25, H 5.49, N 4.01. Found: C 60.02, H, 5.51, N 3.91.

Computational methods for 8 and [Pt(C^N^N)(C≡CPh)]

The hybrid density functional, PBE0,[2] was employed for all calculations using the program package G09.[3] The 6-31G* basis set[4-5] is used for all atoms except Pt, which is described by the Stuttgart relativistic pseudopotential and its accompanying basis set (ECP60MWB).[6-7] Solvent effect was also included by means of the polarizable continuum model (PCM).[8] Geometry optimizations of the singlet ground state (S_0) and the lowest triplet excited state (T_1) were carried out using restricted and unrestricted density functional theory (i.e. RDFT and UDFT) formalism respectively without symmetry constraints. Frequency calculations were performed on the optimized structures to ensure that they are minimum energy structures by the absence of imaginary frequency (i.e. NImag = 0). Stability calculations were also performed for all the optimized structures to ensure that all the wavefunctions obtained are stable. Electron difference density maps (eddms) were created by GaussView5.0. Calculation methods of intramolecular reorganization energy (λ), radiative and non-radiative decay rates were reported elsewhere and were also applied here.[9-10]

Computational results and discussion for 8 and [Pt(C^N^N)(C≡CPh)]

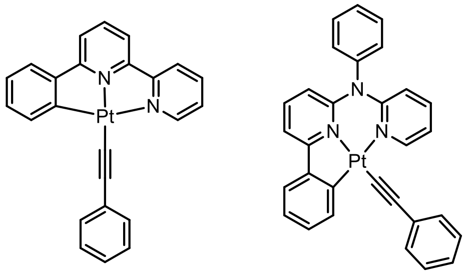

[Pt(C^N^N)(C≡CPh)] **8**

Chart S1 Chemical structures of [Pt(C^N^N)(C≡CPh)] and **8**.

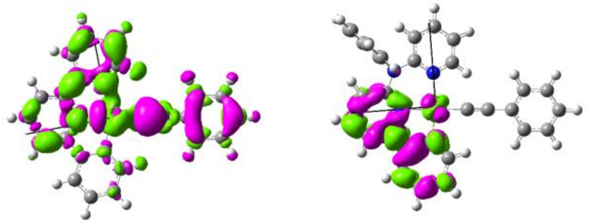

[Pt(C^N^N)(C≡CPh)] **8**

Fig. S1 Electron difference density maps (eddms) of the T_1 excited state for complexes [Pt(C^N^N)(C≡CPh)] and **8**. Magenta: decrease in electron density; moss green: increase in electron density.

There are two critical factors that determine whether k_{nr} is slow or fast: the energy gap (ΔE) and the structural distortion between the T_1 and S_0 states. The energy gap law states that the smaller the energy gap, the faster would be the non-radiative decay rate. So, from the energy gap alone, it is not surprising that [Pt(C^N^N)(C≡CPh)] has a faster k_{nr} than **8** because the former is ~3000 cm^{-1} lower in energy than the latter (calculation predicts that the difference in ΔE is ~3500 cm^{-1}). As was pointed out by Huo *et. al.*, changing the cyclometalated ligand from C^N^N to C^N*N should not increase the rigidity of the Pt(II) complex; the present computational studies actually suggest that **8** has larger intramolecular reorganization energy (λ ~2100 cm^{-1} and ~2800 cm^{-1} for complex [Pt(C^N^N)(C≡CPh)] and **8** respectively) and hence larger structural distortion. However, because the emitting T_1 excited state of [Pt(C^N^N)(C≡CPh)] is of ^{3}LLCT

character, $\omega_{C\equiv C}$ ($\hbar\omega_{C\equiv C}$ ~2200 cm^{-1}), the C≡C stretching normal mode could also act as an effective accepting mode in [Pt(C^N^N)(C≡CPh)], besides the C=C/C=N aromatic stretching modes (denoted $\hbar\omega_M$ ~1300 − 1400 cm^{-1}). On the other hand, the T_1 excited state of **8** is of $^3\pi\pi^*$(C^N) character and little C≡C distortion is involved; hence, only C=C/C=N aromatic stretching modes could act as the effective accepting modes in the non-radiative T_1 → S_0 transition. Therefore, as [Pt(C^N^N)(C≡CPh)] has a smaller energy gap and two high-frequency accepting modes, a smaller number of accepting modes are required and so, [Pt(C^N^N)(C≡CPh)] has a faster non-radiative decay rate. The present computational studies reveal that the k_{nr} of [Pt(C^N^N)(C≡CPh)] is ~127 times faster than that of **8**, in reasonable agreements with the experimental trend (k_{nr} of complex [Pt(C^N^N)(C≡CPh)] is ~50 times faster than that of **8**).

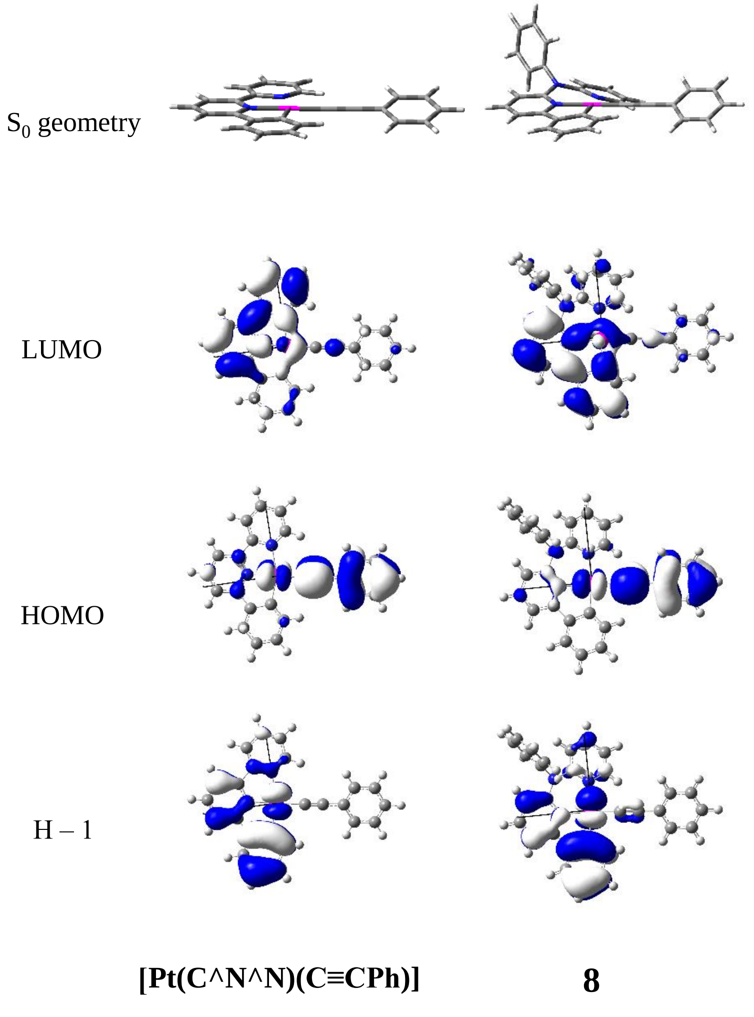

Fig. S2 Optimized S_0 geometries and MO surfaces (S_0) for [Pt(C^N^N)(C≡CPh)] and **8**.

The two lowest-lying singlet excited states of the two complexes are mainly of HOMO → LUMO (S_1) and H−1 → LUMO (S_2) transitions. As seen from Fig. S2, the two occupied orbitals are of similar characters for the two complexes: H−1 is mainly composed of Pt(d) and π(C^N) (C^N is the phenylpyridyl part of the cyclometalated ligand of C^N^N or C^N*N) and HOMO is Pt(d) and π(C≡CPh) but the unoccupied orbitals are of different character: LUMO of

[Pt(C^N^N)(C≡CPh)] is localized on the π*(N^N) part of the C^N^N cyclometalated ligand while that of **8** is localized on π*(C^N) part of the C^N*N cyclometalated ligand. In effect, S_1 of both complexes are of charge-transfer type, ^{1}LLCT; however, S_2 of [Pt(C^N^N)(C≡CPh)] is also of charge transfer type, ^{1}ILCT (ILCT = intraligand charge transfer) but that of **8** is a localized ^{1}ππ*(C^N) (both perturbed with ^{1}MLCT). As the singlet-triplet energy gap of a charge transfer excited state is smaller than that of a localized excited state, T_1 of [Pt(C^N^N)(C≡CPh)] is ^{3}LLCT but that of **8** is ^{3}ππ*(C^N).

The localization of the LUMO on the C^N moiety in **8** instead of N*N moiety as happened in [Pt(C^N^N)(C≡CPh)] could readily be understood by noting their optimized S_0 (or T_1) states. For [Pt(C^N^N)(C≡CPh)], all the three moieties, phenyl and the two pyridyl moieties, in the C^N^N cyclometalated ligand are coplanar but for **8**, the additional *N*-phenyl bridge between the C^N and pyridyl moieties causes the C^N*N ligand to be non-coplanar (see Fig. S2).

Table S1 Optimized S_0 geometry of [Pt(C^N^N)(C≡CPh)]

Center Number	Atomic Number	Atomic Type	Coordinates (Angstroms) X	Y	Z
1	78	0	0.002121	0.002528	-0.009236
2	7	0	-0.005956	0.016594	2.120330
3	7	0	1.955312	0.001954	0.399008
4	6	0	-1.069894	0.022463	2.928803
5	1	0	-2.033545	0.021012	2.429374
6	6	0	-0.952196	0.029872	4.313097
7	1	0	-1.843882	0.034359	4.930189
8	6	0	0.320124	0.031108	4.872643
9	1	0	0.452169	0.036697	5.949910
10	6	0	1.430017	0.024890	4.034515
11	1	0	2.430246	0.025482	4.452344
12	6	0	1.244735	0.017571	2.655474
13	6	0	2.357404	0.009889	1.682080
14	6	0	3.716839	0.009813	1.978420
15	1	0	4.070196	0.016072	3.002714
16	6	0	4.622635	0.001319	0.918272
17	1	0	5.688236	0.001095	1.125457
18	6	0	4.177287	-0.006806	-0.398841
19	1	0	4.887745	-0.013217	-1.217821
20	6	0	2.803404	-0.006319	-0.651761
21	6	0	2.102911	-0.013346	-1.941217
22	6	0	0.673471	-0.010525	-1.878476
23	6	0	-0.018040	-0.016521	-3.094179
24	1	0	-1.104352	-0.014741	-3.081669
25	6	0	0.658260	-0.024911	-4.315255
26	1	0	0.091443	-0.029273	-5.243561
27	6	0	2.052288	-0.027666	-4.356719

28	1	0	2.574421	-0.034192	-5.309275
29	6	0	2.773610	-0.021888	-3.167874
30	1	0	3.860543	-0.023953	-3.201679
31	6	0	-1.908488	0.003120	-0.394120
32	6	0	-3.124896	0.003704	-0.585784
33	6	0	-4.530755	0.001907	-0.824249
34	6	0	-5.440907	0.307560	0.205536
35	1	0	-5.056652	0.547671	1.193109
36	6	0	-6.810652	0.305344	-0.031059
37	1	0	-7.495667	0.545303	0.778133
38	6	0	-7.306871	-0.003166	-1.297362
39	1	0	-8.377998	-0.005130	-1.479984
40	6	0	-6.417990	-0.309113	-2.327529
41	1	0	-6.795344	-0.550859	-3.317862
42	6	0	-5.047126	-0.306372	-2.097432
43	1	0	-4.356478	-0.544483	-2.901599

Table S2 Optimized T_1 geometry of [Pt(C^N^N)(C≡CPh)]

Center Number	Atomic Number	Atomic Type	Coordinates (Angstroms) X	Y	Z
1	78	0	0.000000	0.000000	0.000000
2	7	0	0.000000	0.000000	2.112657
3	7	0	1.936415	0.000000	0.371997
4	6	0	-1.056642	0.000901	2.945434
5	1	0	-2.027770	0.001823	2.459085
6	6	0	-0.935596	0.001202	4.318485
7	1	0	-1.823209	0.001795	4.941237
8	6	0	0.360732	-0.000388	4.877063
9	1	0	0.496002	-0.000941	5.954457
10	6	0	1.454656	-0.001347	4.042911
11	1	0	2.458373	-0.002594	4.455573
12	6	0	1.282241	-0.000972	2.642308
13	6	0	2.349817	-0.001262	1.689049
14	6	0	3.732776	-0.002773	1.941219
15	1	0	4.102975	-0.006370	2.961076
16	6	0	4.617533	0.000487	0.880157
17	1	0	5.685965	-0.000112	1.073482
18	6	0	4.153917	0.005279	-0.459995
19	1	0	4.859921	0.009405	-1.282901
20	6	0	2.797375	0.004958	-0.693129
21	6	0	2.080615	0.010742	-1.974276
22	6	0	0.653646	0.009252	-1.896194
23	6	0	-0.061805	0.016280	-3.097394

24	1	0	-1.148453	0.016539	-3.070097
25	6	0	0.591712	0.024267	-4.330814
26	1	0	0.009404	0.030187	-5.249384
27	6	0	1.985124	0.025335	-4.391804
28	1	0	2.492921	0.031795	-5.352354
29	6	0	2.725925	0.018450	-3.214673
30	1	0	3.812273	0.019465	-3.265163
31	6	0	-1.852633	0.046850	-0.366921
32	6	0	-3.090240	0.094783	-0.573534
33	6	0	-4.464018	0.145130	-0.820870
34	6	0	-5.395733	0.195612	0.248429
35	1	0	-5.023397	0.203314	1.268039
36	6	0	-6.753552	0.234516	-0.012496
37	1	0	-7.462587	0.273056	0.808924
38	6	0	-7.214137	0.223975	-1.333754
39	1	0	-8.281451	0.254535	-1.532873
40	6	0	-6.309802	0.173979	-2.400446
41	1	0	-6.676448	0.165905	-3.422445
42	6	0	-4.949184	0.134956	-2.154353
43	1	0	-4.235675	0.095780	-2.971562

Table S3 Optimized S_0 geometry of **8**

Center Number	Atomic Number	Atomic Type	Coordinates (Angstroms) X	Y	Z
1	78	0	-0.119282	-0.012993	0.078981
2	7	0	-0.075380	0.137062	2.124700
3	7	0	2.308944	0.054195	2.308179
4	7	0	1.937771	0.501100	-0.021211
5	6	0	-2.057901	-0.259140	0.406833
6	6	0	-3.091132	-0.524962	-0.502901
7	1	0	-2.844474	-0.685943	-1.547830
8	6	0	-4.421930	-0.577801	-0.093529
9	1	0	-5.201550	-0.787694	-0.822526
10	6	0	-4.762773	-0.359283	1.242300
11	1	0	-5.801365	-0.394393	1.559243
12	6	0	-3.762255	-0.099667	2.169050
13	1	0	-4.034459	0.065307	3.208165
14	6	0	-2.421538	-0.054918	1.763487
15	6	0	-1.317434	0.151800	2.701154
16	6	0	-1.459567	0.285186	4.075846
17	1	0	-2.444191	0.299497	4.526474
18	6	0	-0.319982	0.379379	4.862439
19	1	0	-0.402928	0.495052	5.938816

20	6	0	0.930612	0.314558	4.276498
21	1	0	1.820940	0.368712	4.885987
22	6	0	1.031604	0.184598	2.882690
23	6	0	2.758654	0.478552	1.046322
24	6	0	4.108174	0.854188	0.911564
25	1	0	4.763575	0.827323	1.770457
26	6	0	4.594512	1.272178	-0.310797
27	1	0	5.634709	1.569510	-0.402785
28	6	0	3.731605	1.321539	-1.403021
29	1	0	4.058766	1.647364	-2.383891
30	6	0	2.424164	0.934070	-1.207183
31	1	0	1.705951	0.926707	-2.018491
32	6	0	3.307229	-0.558531	3.155840
33	6	0	4.096208	0.197959	4.024352
34	1	0	3.965590	1.275986	4.072670
35	6	0	5.045123	-0.437797	4.820391
36	1	0	5.659268	0.149851	5.496346
37	6	0	5.205751	-1.820861	4.749016
38	1	0	5.947208	-2.313262	5.371452
39	6	0	4.416319	-2.570481	3.879913
40	1	0	4.538794	-3.647979	3.821394
41	6	0	3.464716	-1.940544	3.081209
42	1	0	2.841545	-2.511705	2.398807
43	6	0	-0.301233	-0.228295	-1.843472
44	6	0	-0.398904	-0.372720	-3.061303
45	6	0	-0.520444	-0.539706	-4.472702
46	6	0	-1.728322	-0.246326	-5.133746
47	1	0	-2.573274	0.111900	-4.552100
48	6	0	-1.843640	-0.408726	-6.509647
49	1	0	-2.785364	-0.175809	-7.000111
50	6	0	-0.761005	-0.867753	-7.259343
51	1	0	-0.853949	-0.994519	-8.334508
52	6	0	0.441694	-1.163243	-6.618201
53	1	0	1.291118	-1.522476	-7.193640
54	6	0	0.563747	-1.001603	-5.242833
55	1	0	1.501647	-1.233667	-4.745727

Table S4 Optimized T_1 geometry of **8**

Center Number	Atomic Number	Atomic Type	Coordinates (Angstroms) X	Y	Z
1	78	0	-0.133272	0.019058	0.082543
2	7	0	-0.057313	0.195263	2.097793
3	7	0	2.308472	0.060217	2.298341

4	7	0	1.936046	0.532640	-0.024185
5	6	0	-2.045647	-0.229550	0.400547
6	6	0	-3.082865	-0.508538	-0.492445
7	1	0	-2.839457	-0.670874	-1.537761
8	6	0	-4.409472	-0.575163	-0.075854
9	1	0	-5.198156	-0.793573	-0.790150
10	6	0	-4.745499	-0.351489	1.293908
11	1	0	-5.787468	-0.401458	1.599541
12	6	0	-3.781816	-0.080530	2.221000
13	1	0	-4.060088	0.080911	3.257761
14	6	0	-2.399842	-0.015171	1.822886
15	6	0	-1.346582	0.221479	2.698795
16	6	0	-1.461902	0.380236	4.117662
17	1	0	-2.446099	0.437410	4.568019
18	6	0	-0.336202	0.436216	4.883950
19	1	0	-0.396435	0.557036	5.960607
20	6	0	0.933687	0.332359	4.267692
21	1	0	1.830910	0.370229	4.867139
22	6	0	1.027719	0.205987	2.862761
23	6	0	2.761867	0.489551	1.039138
24	6	0	4.113301	0.849869	0.900923
25	1	0	4.773645	0.803199	1.755373
26	6	0	4.597228	1.275768	-0.321412
27	1	0	5.640725	1.560080	-0.416920
28	6	0	3.729134	1.348512	-1.407140
29	1	0	4.054437	1.681894	-2.386040
30	6	0	2.417480	0.973065	-1.207684
31	1	0	1.694181	0.981615	-2.014530
32	6	0	3.291496	-0.583419	3.138802
33	6	0	4.105756	0.147799	4.005990
34	1	0	4.002236	1.228376	4.061184
35	6	0	5.040747	-0.516887	4.794614
36	1	0	5.673438	0.050783	5.470572
37	6	0	5.163674	-1.903527	4.716609
38	1	0	5.894742	-2.418353	5.333196
39	6	0	4.349808	-2.627978	3.848657
40	1	0	4.442695	-3.708163	3.785261
41	6	0	3.411623	-1.969069	3.057512
42	1	0	2.769611	-2.520072	2.375939
43	6	0	-0.311203	-0.215690	-1.845781
44	6	0	-0.397634	-0.368471	-3.063602
45	6	0	-0.505525	-0.546296	-4.474056
46	6	0	-1.754889	-0.468533	-5.118788
47	1	0	-2.642425	-0.268505	-4.524831
48	6	0	-1.857322	-0.643114	-6.494012
49	1	0	-2.831667	-0.579083	-6.971702

50	6	0	-0.720023	-0.898915	-7.259702
51	1	0	-0.802905	-1.035298	-8.334497
52	6	0	0.524453	-0.978636	-6.635082
53	1	0	1.416584	-1.178235	-7.223244
54	6	0	0.633601	-0.804804	-5.260320
55	1	0	1.604069	-0.869385	-4.775903

Computational methods for dispersion interaction on dimers of [61–PF$_6$]$^{2+}$

All calculations were performed with the program package G09. Starting geometry was directly extracted from the CCDC crystal structure database. Hybrid functional PBE0 with dispersion corrections in revision three (D3)[11] was employed for its accuracy to describe dispersion interaction. Since basis sets of at least triple-ζ quality with diffuse functions[12] are recommended for calculating noncovalent systems, the valence atomic orbitals of platinum were described by LANL2TZ basis set with one f diffuse function. The effective core potentials (ECPs) proposed by Hay and Wadt were employed on platinum atom.[13-14] The 6-31+G* basis set was used for hydrogen, carbon and nitrogen atoms. The potential energy surface (PES) of **[61–PF$_6$]$^{2+}$** was constructed by single-point DFT calculations, using M-M' distance as a reaction coordinate. Solvent effects were taken into account by the polarizable continuum model (PCM) with acetonitrile as solvent.

Table S5 Crystal data of complexes **68** (monoclinic) and **68** (triclinic)

	**68** (monoclinic)	**68** (triclinic)
formula	$C_{35}H_{38}N_2OPt$	$C_{35}H_{38}N_2OPt$•$C_{35}H_{38}N_2OPt$
Mr	697.75	1393.49
Color	Red	Yellow
crystal size/mm^3	0.06×0.04×0.04	0.02×0.01×0.01
crystal system	Monoclinic	Triclinic
space group	P 2_1/n	P$\bar{1}$
a/Å	9.3787(6)	12.877(3)
b/Å	15.5048(9)	12.914(3)
c/Å	19.8579(12)	18.125(4)
α/°	90	78.472(11)
β/°	103.130(2)	76.592(12)
γ/°	90	79.837(10)
V / Å^3	2812.1(3)	2845.9(11)
Z	4	2
D_c, g cm^{-3}	1.648	1.626
μ, cm^{-1}	9.562	9.448
F(000)	1392.0	1388.0
$2\theta_{max}$, deg	133.56	132.76
residual ρ, e Å^{-3}	4.985, -6.931	3.25, -1.49
for $I > 2\sigma(I)$		
R^a	0.0180	0.0964
Rw^b	0.0472	0.2763

[a] $R = \Sigma \,||F_o| - |F_c|| / \Sigma |F_o|$. [b] $R_w = [\Sigma w (|F_o| - |F_c|)^2 / \Sigma w |F_o|^2]^{1/2}$

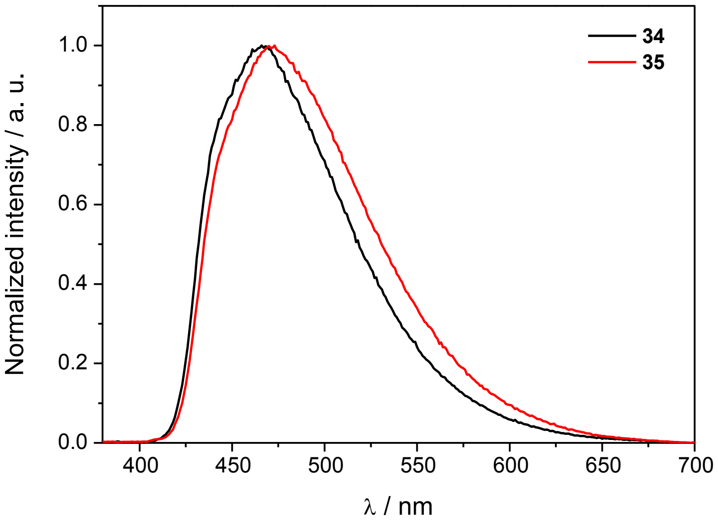

Fig. S3 Emission spectra of **34** and **35** in THF.

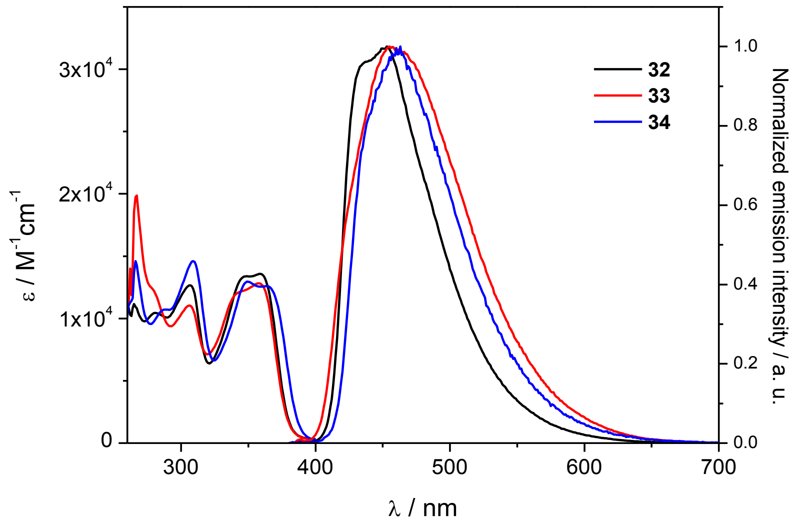

Fig. S4 Absorption and emission spectra of **32–34** in DMF.

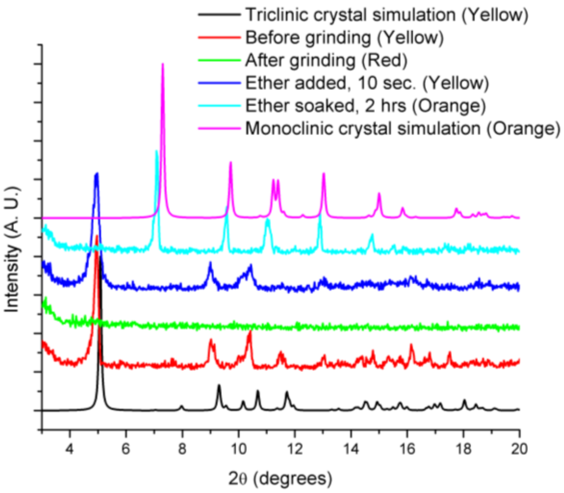

Fig. S5 Powder XRD diffractograms of **68** in different crystal/solid forms.

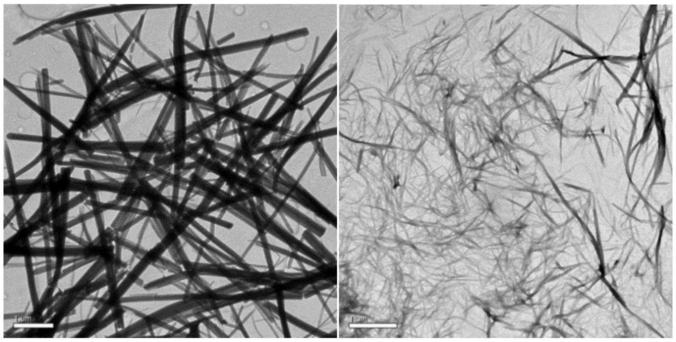

Fig. S6 TEM images of the aggregates of **70** obtained from (Left) CH_2Cl_2/hexane (v/v, 1:1) or (Right) DMSO/H_2O (v/v, 1:9).

References

1 R. Tanaka, A. Rubio, N. K. Harn, D. Gernert, T. A. Grese, J. Eishima, M. Hara, N. Yoda, R. Ohashi, T. Kuwabara, S. Soga, S. Akinaga, S. Nara and Y. Kanda, *Biorg. Med. Chem.*, 2007, **15**, 1363.

2 C. Adamo and V. Barone, *J. Chem. Phys.*, 1999, **110**, 6158.

3 M. J. Frisch, G. W. Trucks, H. B. Schlegel, G. E. Scuseria, M. A. Robb, J. R. Cheeseman, G. Scalmani, V. Barone, B. Mennucci, G. A. Petersson, H. Nakatsuji, M. Caricato, X. Li, H. P. Hratchian, A. F. Izmaylov, J. Bloino, G. Zheng, J. L. Sonnenberg, M. Hada, M. Ehara, K. Toyota, R. Fukuda, J. Hasegawa, M. Ishida, T. Nakajima, Y. Honda, O. Kitao, H. Nakai, T. Vreven, J. A. Montgomery Jr, J. E. Peralta, F. Ogliaro, M. Bearpark, J. J. Heyd, E. Brothers, K. N. Kudin, V. N. Staroverov, R. Kobayashi, J. Normand, K. Raghavachari, A. Rendell, J. C. Burant, S. S. Iyengar, J. Tomasi, M. Cossi, N. Rega, J. M. Millam, M. Klene, J. E. Knox, J. B. Cross, V. Bakken, C. Adamo, J. Jaramillo, R. Gomperts, R. E. Stratmann, O. Yazyev, A. J. Austin, R. Cammi, C. Pomelli, J. W. Ochterski, R. L. Martin, K. Morokuma, V. G. Zakrzewski, G. A. Voth, P. Salvador, J. J. Dannenberg, S. Dapprich, A. D. Daniels, Ö. Farkas, J. B. Foresman, J. V. Ortiz, J. Cioslowski and D. J. Fox, Gaussian, Inc., Wallingford CT, 2009.

4 M. M. Francl, W. J. Petro, W. J. Hehre, J. S. Binkley, M. S. Gordon, D. J. DeFree and J. A. Pople, *J. Chem. Phys.*, 1982, **77**, 3654.

5 P. C. Hariharan and J. A. Pople, *Theor. Chim. Acta*, 1973, **28**, 213.

6 D. Andrae, U. Haeussermann, M. Dolg, H. Stoll and H. Preuss, *Theor. Chim. Acta*, 1990, **77**, 123.

7 J. M. L. Martin and A. Sundermann, *J. Chem. Phys.*, 2001, **114**, 3408.

8 M. Cossi, G. Scalmani, N. Rega and V. Barone, *J. Chem. Phys.*, 2002, **117**, 43.

9 G. S. M. Tong, K. T. Chan, X. Chang and C.-M. Che, *Chem. Sci.*, 2015, **6**, 3026.

10 G. S. M. Tong, P. K. Chow, W.-P. To, W.-M. Kwok and C.-M. Che, *Chem. Eur. J.*, 2014, **20**, 6433.

11 S. Grimme, J. Antony, S. Ehrlich and H. Krieg, *J. Chem. Phys.*, 2010, **132**, 154104.
12 P. Jurečka, J. Černý, P. Hobza and D. R. Salahub, *J. Comput. Chem.*, 2007, **28**, 555.
13 W. R. Wadt and P. J. Hay, *J. Chem. Phys.*, 1985, **82**, 284.
14 P. J. Hay and W. R. Wadt, *J. Chem. Phys.*, 1985, **82**, 299.
